# Transcriptome analysis of *Zymomonas mobilis* ZM4 reveals mechanisms of tolerance and detoxification of phenolic aldehyde inhibitors from lignocellulose pretreatment

**DOI:** 10.1186/s13068-015-0333-9

**Published:** 2015-09-22

**Authors:** Xia Yi, Hanqi Gu, Qiuqiang Gao, Z. Lewis Liu, Jie Bao

**Affiliations:** State Key Laboratory of Bioreactor Engineering, East China University of Science and Technology, 130 Meilong Road, Shanghai, 200237 China; US Department of Agriculture, Agricultural Research Service, National Center for Agricultural Utilization Research, 1815 North University Street, Peoria, IL 61604 USA

**Keywords:** *Zymomonas mobilis* ZM4, Lignocellulose pretreatment, Phenolic aldehyde inhibitors, Stress tolerance, DNA microarray, Recombinants

## Abstract

**Background:**

Phenolic aldehydes generated from lignocellulose pretreatment exhibited severe toxic inhibitions on microbial growth and fermentation. Numerous tolerance studies against furfural, 5-hydroxymethyl-2-furaldehyde (HMF), acetate, and ethanol were reported, but studies on inhibition of phenolic aldehyde inhibitors are rare. For ethanologenic strains, *Zymomonas mobilis* ZM4 is high in ethanol productivity and genetic manipulation feasibility, but sensitive to phenolic aldehyde inhibitors. Molecular mechanisms of tolerance for *Z. mobilis* toward phenolic aldehydes are not known.

**Results:**

We took the first insight into genomic response of *Z. mobilis* ZM4 to the phenolic aldehyde inhibitors derived from lignocellulose pretreatment. The results suggest that the toxicity to cells is caused by the functional group of phenolic aldehyde, similar to furfural and HMF, rather than aromatic groups or phenolic hydroxyl groups. Transcriptome response against 4-hydroxybenzaldehyde, syringaldehyde, and vanillin, representing phenolic groups H, S, and G, respectively, was investigated. The atlas of the important genes responsible for significantly enhanced and repressed genes at the genomic level was illustrated. 272 genes with twofold greater expressions than non-treated controls and 36 gene clusters in response to challenges of these phenolic aldehydes were identified. Several reductases encoded by *ZMO1116*, *ZMO1696*, and *ZMO1885* were found to play the key roles in reducing phenolic aldehydes into the corresponding phenolic alcohols. Reduction of phenolic aldehydes by overexpression of *ZMO1116*, *ZMO1696*, and *ZMO1885* in *Z. mobilis* ZM4 resulted in the increased inhibitor conversion and ethanol productivity, especially for 4-hydroxybenzaldehyde and vanillin. Several transporter genes such as *ZMO0282*, *ZMO0283*, *ZMO0798*, *ZMO0799*, and *ZMO0800* was also displayed significantly increased expressions against the phenolic aldehydes.

**Conclusions:**

The genes encoding reductases are with potentials on phenolic aldehydes-tolerant genes contributing to the reduction of phenolic aldehydes into the corresponding phenolic alcohols forms for *Z. mobilis* ZM4. Overexpression of the key genes improved the conversion ratio and ethanol productivity of 4-hydroxybenzaldehyde and vanillin with high toxicity. New knowledge obtained from this research aids understanding the mechanisms of bacterial tolerance and the development of the next-generation biocatalysts for advanced biofuels production.

**Electronic supplementary material:**

The online version of this article (doi:10.1186/s13068-015-0333-9) contains supplementary material, which is available to authorized users.

## Background

Pretreatment is the central step for releasing fermentable sugars from lignocellulose biomass. During harsh pretreatment processes, various small molecules are generated from partial over-degradation of lignocellulose and inhibit consequent microbial fermentations. Commonly observed inhibitory compounds include furan aldehydes such as 2-furylaldehyde (furfural) and 5-hydroxymethyl-2-furaldehyde (HMF) from dehydration of pentoses and hexoses, weak organic acids such as acetic acid, formic acid, and levulinic acid from carboxylate group hydrolysis or furans oxidations, as well as phenolic compounds degraded from partial breakdown of lignin components [1, 2]. Phenolic compounds are classified into three major groups according to their methoxyl and functional groups: (1) *p*-hydroxyphenyl group (H) represented by 4-hydroxybenzaldehyde, 4-hydroxybenzoate, and 4-hydroxybenzyl alcohol; (2) syringyl group (S) including syringaldehyde (4-hydroxy-3,5-dimethoxybenzaldehyde), syringate, and syringic alcohol; and (3) guaiacyl group (G) including guaiacol, vanillin (4-hydroxy-3-methoxybenzaldehyde), vanillate, vanillyl alcohol, ferulic acid, and coniferyl aldehyde. Most of these phenolic compounds had been recognized in pretreated lignocellulose materials, including 4-hydroxybenzaldehyde, 4-hydroxybenzoate, syringaldehyde, syringate, vanillin, vanillate, and coniferyl aldehyde [1]. Phenolic compounds showed severe toxic inhibitions on fermenting strains [3] and cellulase activities [4]. Yet, identification and accurate measurement of all phenolic compounds in lignocellulose hydrolysate remain challenging because of poor water solubility and large number of derivatives.

Ethanologenic strain *Zymomonas mobilis* ZM4 has demonstrated its potential in lignocellulose biorefinery applications for high ethanol productivity, ethanol tolerance, and genetic manipulation feasibility [5, 6]. It tolerates high levels of phenolic acids [7], but is sensitive to phenolic aldehydes. Existence of minimum vanillin and syringaldehyde will lead to 1/3 decrease of ethanol yield for *Z. mobilis* [8] due to the disruptions of cell membrane and enzyme hydrophobic sites by phenolic compounds [1, 2]. While numerous studies on tolerance of varied strains were reported against ethanol [9], acetate [10], furfural [11], studies on inhibition of phenolic aldehyde inhibitors are rare. Molecular mechanisms of tolerance for *Z. mobilis* toward phenolic aldehydes are not known.

In this study, we investigated the profiles of genome expression of *Z. mobilis* ZM4 using DNA microarray in response to three typical phenolic aldehydes, 4-hydroxybenzaldehyde representing phenolic group H, syringaldehyde for group S, and vanillin for group G. Candidate genes against varied phenolic aldehydes were identified from *Z. mobilis* ZM4 and were selectively expressed in *Z. mobilis* ZM4 for the confirmation of gene functions. This study provides the first insight into the genome response of *Z. mobilis* against phenolic aldehyde inhibitors. Tolerant genes identified in this study will serve as valuable resources for robust strain development for future biorefinery applications.

## Results

### Cell growth and fermentation response of *Z. mobilis* ZM4 to phenolic aldehydes

**Fig. 1:**
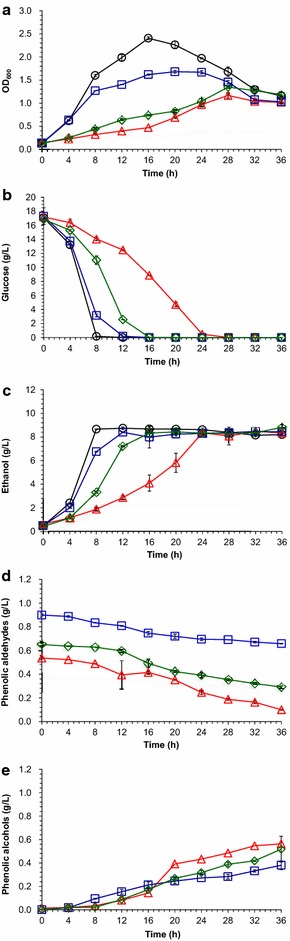
Inhibition of performance for *Z. mobilis* strain ZM4 by selective phenolic aldehydes. **a** Cell growth as measured by optical density at 600 nm (OD_600nm_); **b** glucose consumption; **c** ethanol fermentation; **d** degradation of phenolic aldehydes; **e** formation of phenolic alcohols in RM medium separately amended with 5 mM of 4-hydroxybenzaldehyde, syringaldehyde, and vanillin

The maximum cell growth of *Z. mobilis* ZM4 was depressed approximately 5.0, 1.4, and 3.1-folds when 4-hydroxybenzaldehyde, syringaldehyde, and vanillin were separately added, comparing to the non-phenolic aldehydes-treated culture (Fig. 1a). *Z. mobilis* ZM4 completely consumed glucose and reached the maximum ethanol titer at 8 h, but delayed about 12, 16, and 24 h and led to decrease of ethanol productivity from 0.73 to 0.24, 0.70, and 0.61 g/L/h when 4-hydroxybenzaldehyde, syringaldehyde, and vanillin were separately added (Fig. 1b, c). Apparently, 4-hydroxybenzaldehyde is the most potent inhibitory compound to *Z. mobilis* ZM4 among the three evaluated phenolic aldehydes. Conversion of phenolic aldehydes into the corresponding phenolic alcohols by *Z. mobilis* ZM4 was identified by GC–MS (Fig. 1d, e, Additional file 1). No further degradation of phenolic alcohols to other metabolites was detected. Approximately, 80 % of 4-hydroxybenzaldehyde was reduced into 4-hydroxybenzyl alcohol and 70 % of vanillin degraded into vanillyl alcohol after incubated for 36 h. On the other hand, syringaldehyde was relatively stable with only a minimum degradation in 36 h. The results were partially in agreement with the previous studies of *Z. mobilis* ZM4 converting 4-hydroxybenzaldehyde, syringaldehyde, and vanillin into their alcoholic forms [12].

### Transcriptome response of *Z. mobilis* ZM4 against phenolic aldehydes

**Fig. 2:**
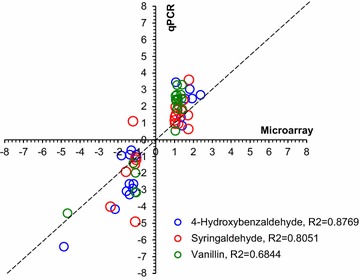
Comparison of gene expression levels of *Z. mobilis* ZM4 between the DNA microarray and qRT-PCR. The gene expression ratios of both microarray data and qRT-PCR data for 20 genes were log transformed in base 2 (log_2_, treatment/control), and the microarray log_2_ ratio values were plotted against the qRT-PCR log_2_ values. The 20 selected genes included *ZMO0152* (pyruvate kinase), *ZMO0177* (16S rRNA), *ZMO0179* (fructose-bisphosphate aldolase), *ZMO0367* (glucose-6-phosphate 1-dehydrogenase), *ZMO0368* (6-phosphogluconate dehydrogenase), *ZMO0369* (glucokinase), *ZMO0387* (hpcH/hpaI aldolase), *ZMO0543* (aconitate hydratase), *ZMO0544* (isocitrate dehydrogenase), *ZMO0567* (succinyl-CoA synthetase), ZMO0569 (succinate dehydrogenase), *ZMO0997* (KDPG aldolase), *ZMO1237* (d-isomer specific 2-hydroxyacid dehydrogenase), *ZMO1307* (fumarase), *ZMO1360* (pyruvate decarboxylase), *ZMO1478* (6-phosphogluconolactonase), *ZMO1496* (phosphoenolpyruvate carboxylase), *ZMO1596* (Fe-containing alcohol dehydrogenase), *ZMO1608* (phosphopyruvate hydratase), and *ZMO1963* (citrate synthase). The primers for qRT-PCR of the genes are listed in Additional file 1

The microarray results revealed that a total of 2020 gene fragments were amplified. To confirm microarray expression data, 20 genes involving in central carbon metabolic pathways (Additional file 2) were selected for quantitative expression using qRT-PCR under the stress of the three phenolic aldehydes. Results were consistent in detection of the trend of up- or down-regulated expressions for both assays, except for only one gene showing a marginal variation (Fig. 2). As anticipated, quantitative results of qRT-PCR were more sensitive in general with greater fold changes than that of microarray.

**Fig. 3:**
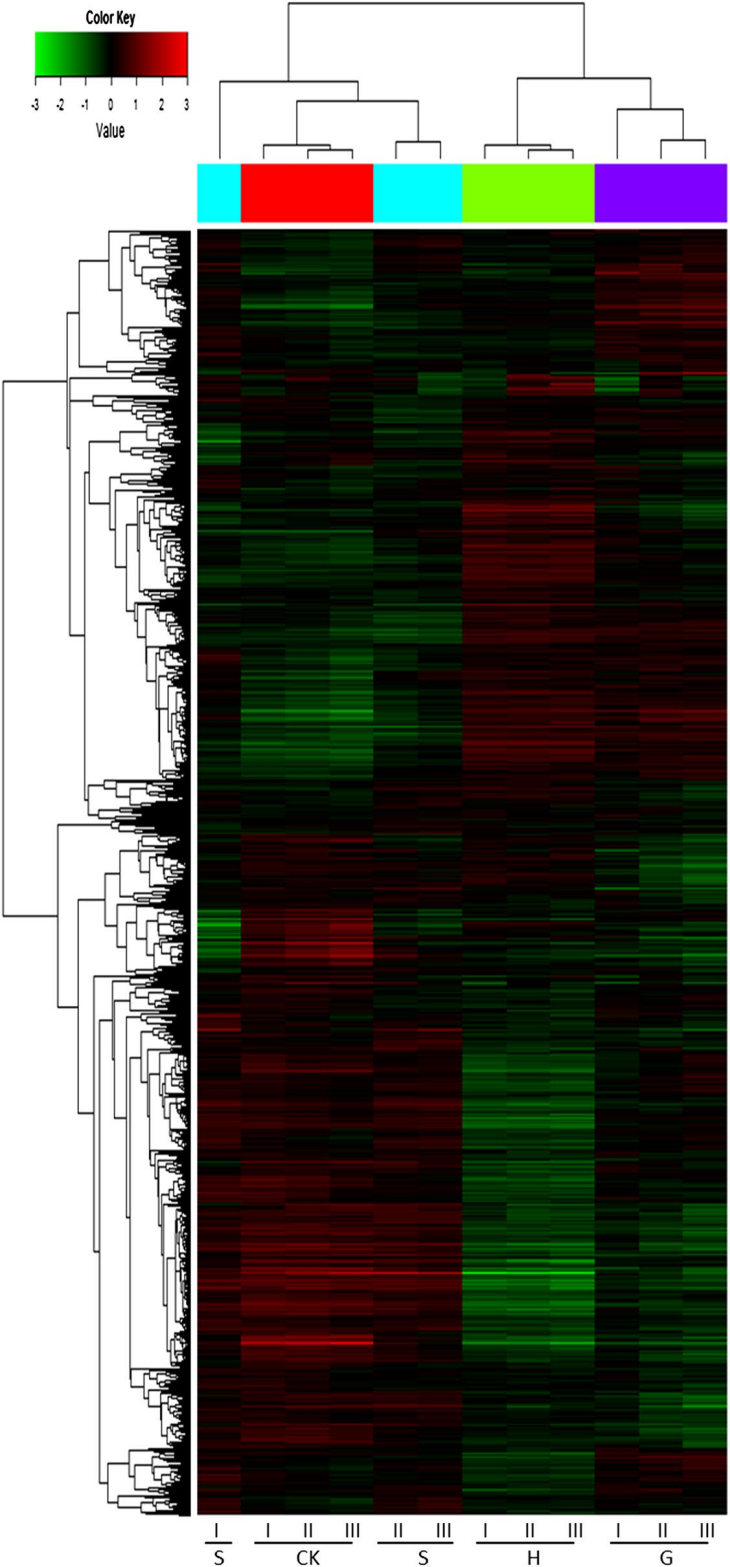
Hierarchical cluster analysis of genome expression of *Z. mobilis* strain ZM4 in response to selective phenolic aldehydes. Gene expression levels were clustered based on log_2_ transformation. Color code *red*, *green*, or *black* each represents up-, down-, or normally regulated expression, respectively. Letter *H*, *G*, and *S*, each stands for, phenolic group H represented by 4-hydroxybenzaldehyde, phenolic group G represented by vanillin, and phenolic group S represented by syringaldehyde, respectively. CK stands for a non-phenolic aldehyde-treated control. Replications of each treatment for the microarray experiment were marked in *Roman numeral*

**Fig. 4:**
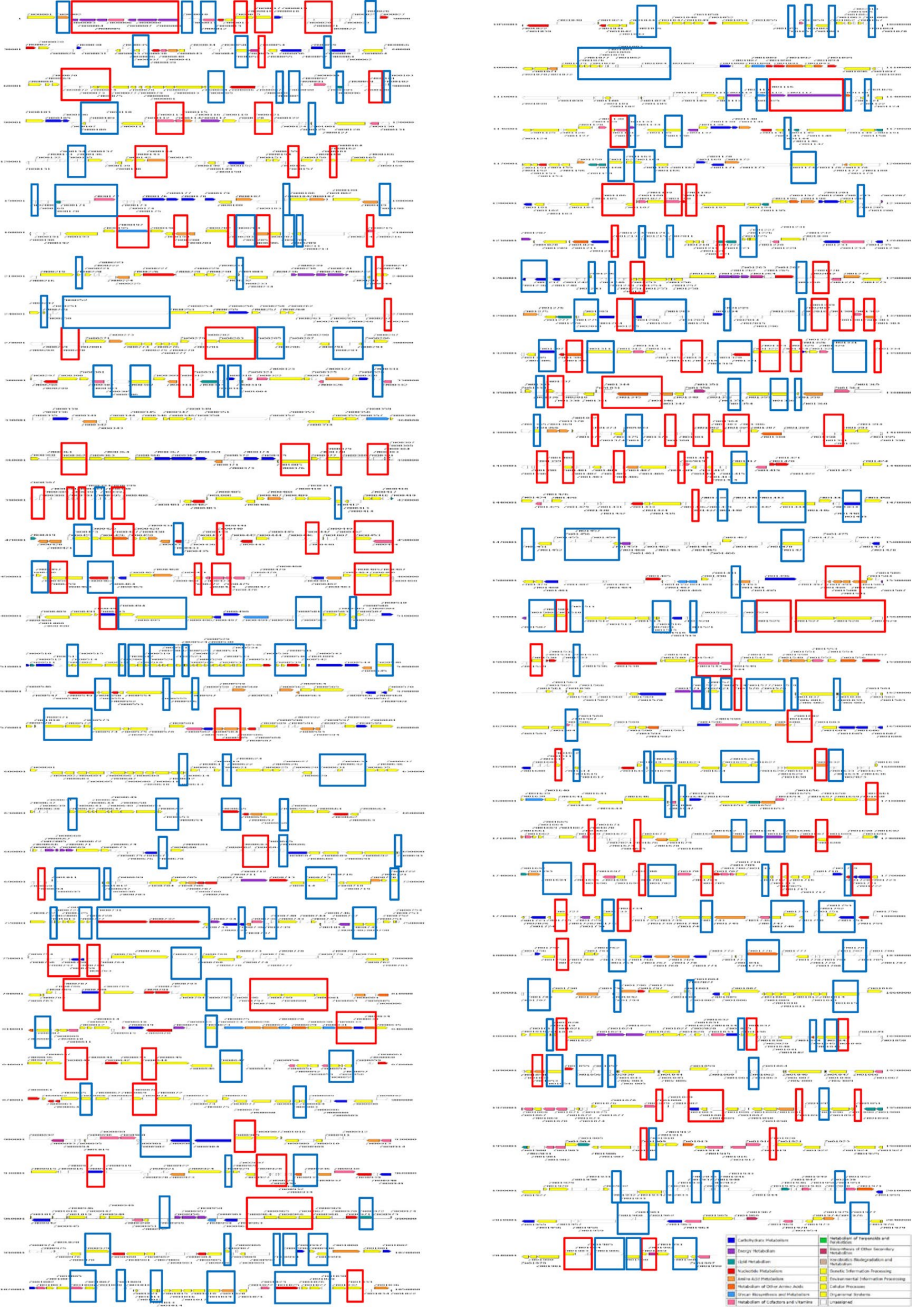
Genome map of *Z. mobilis* strain ZM4 showing expression profiling in response to phenolic aldehyde challenges. Genes and gene clusters shown significantly up-regulated expressions were *boxed* in *red*, and those significantly down-regulated expressions *boxed* in *blue*. The genome map representing 1998 genes was adapted from KEGG database. A *colored code* indicating annotated gene functional categories is also attached

Our hierarchical cluster analysis showed two clear distinguished expression patterns for 1908 genes in the genome of *Z. mobilis* ZM4 in response to challenges of 4-hydroxybenzaldehyde, syringaldehyde, and vanillin representing three groups of phenolic aldehyde inhibitors (Fig. 3). The genome response to syringaldehyde challenge was similar and very closely related to the non-treated control. In contrast, the expression patterns against 4-hydroxybenzaldehyde and vanillin were closely related and distinct from the control as well as the responses treated with syringaldehyde. Results from the replicated microarray experiments showed high levels of consistence with minimum variations within each treatment. Based on their expression responses, two major groups of genes were clustered separately. The control and the syringaldehyde-treated cultures shared a group of approximate 16 genes showing up-regulated response. In contrast, most genes in this group displayed a repressed or down-regulated expression in response to the 4-hydroxybenzaldehyde or vanillin treatment. On the other hand, many genes showing up-regulated expressions against 4-hydroxybenzaldehyde and vanillin displayed normal or down-regulated expressions for the syringaldehyde treated and the control. In response to challenge of a single phenolic aldehyde, 144 genes showed up-regulated expression and 299 genes repressed with a greater than twofold changes against 4-hydroxybenzaldehyde, a representative of *p*-hydroxyphenyl group (H), compared with the control (Additional file 3). For vanillin, a representative of guaiacyl group, 111 genes were up-regulated expressed and 198 genes down-regulated. For syringaldehyde representing syringyl group (S), only 17 genes were up-regulated and 63 down-regulated. We found 272 genes including 36 gene clusters showing up-regulated expressions against these phenolic aldehydes distributed throughout the entire genome of *Z. mobilis* ZM4 (Fig. 4). Another 560 genes including 52 gene clusters were repressed significantly by three phenolic aldehydes. We identified 72 candidate tolerance genes showing significantly up-regulated expressions against challenges of at least two phenolic aldehydes examined in this study (Table 1).

**Table 1:** Tolerance genes of *Z. mobilis* ZM4 against at least two phenolic aldehydes identified by gene expression analysis

Category	Locus	Product	Expression^a^		
			H	G	S
Amino acid metabolisms	*ZMO0200*	Anthranilate phosphoribosyltransferase	2.88	2.38	
Amino acid metabolisms	*ZMO1303*	Pyrroline-5-carboxylate reductase	2.35	2.23	
Amino acid metabolisms	*ZMO1499*	Phosphoribosyl-ATP diphosphatase	3.46	3.91	2.17
Amino acid metabolisms	*ZMO1500*	Imidazole glycerol phosphate synthase subunit HisF	2.92	2.93	
Amino acid metabolisms	*ZMO1501*	1-(5-phosphoribosyl)-5-[(5-phosphoribosylamino) methylideneamino]imidazole-4-carboxamide isomerase	2.30	2.10	
Amino acid metabolisms	*ZMO1502*	Imidazole glycerol phosphate synthase subunit HisH	2.14	2.25	
Carbohydrate metabolism	*ZMO0493*	Glutamine synthetase, type I	2.80	3.21	2.52
Carbohydrate metabolism	*ZMO0759*	Hydroxyacylglutathione hydrolase	2.66	2.66	
Carbohydrate metabolism	*ZMO0788*	Gluconate 2-dehydrogenase	2.73	2.69	
Carbohydrate metabolism	*ZMO0833*	UDP-*N*-acetylenolpyruvoylglucosamine reductase	2.06	2.14	
Energy metabolism	*ZMO0003*	Adenylylsulfate kinase	2.40	2.01	
Energy metabolism	*ZMO0004*	Sulfate adenylyltransferase subunit 1	3.39	2.31	
Energy metabolism	*ZMO0005*	Sulfate adenylyltransferase subunit 2	3.09	2.13	
**Energy metabolism**	***ZMO1116*** ^b^	**Oxidoreductase**	**2.53**	**3.60**	**2.47**
Lipid metabolism	*ZMO1222*	3-oxoacyl-ACP reductase	2.73	2.11	
Metabolism of cofactors and vitamins	*ZMO0006*	Uroporphyrin-III C-methyltransferase	3.09	2.05	
Metabolism of cofactors and vitamins	*ZMO0474*	3,4-dihydroxy-2-butanone 4-phosphate synthase	2.71	2.34	
Metabolism of cofactors and vitamins	*ZMO1132*	Lipoyl synthase	2.17	2.33	
Metabolism of cofactors and vitamins	*ZMO1190*	Bifunctional phosphopantothenoylcysteine decarboxylase/phosphopantothenate synthase	2.42	2.41	
Metabolism of cofactors and vitamins	*ZMO1544*	Cobalt chelatase, pCobS small subunit	2.84	2.01	
Metabolism of other amino acids	*ZMO1309*	Leucyl aminopeptidase	2.33	2.03	
Genetic/environmental/cellular process/organismal systems	*ZMO0074*	Hypothetical protein	5.02	3.61	
***Genetic/environmental/cellular process/organismal systems***	***ZMO0143*** ^c^	***ATP*** **-** ***binding cassette superfamily***	***2.11***	***2.00***	
***Genetic/environmental/cellular process/organismal systems***	***ZMO0282***	***RND family efflux transporter subunit MFP***	***6.05***	***7.14***	***2.55***
***Genetic/environmental/cellular process/organismal systems***	***ZMO0283***	***Hydrophobe/amphiphile efflux*** **-** ***1 (HAE1) family transporter***	***5.92***	***6.59***	***2.63***
Genetic/environmental/cellular process/organismal systems	*ZMO0472*	rpsU-divergently transcribed protein	3.03	2.55	
Genetic/environmental/cellular process/organismal systems	*ZMO0486*	Hypothetical protein	2.76	2.41	
***Genetic/environmental/cellular Process/organismal systems***	***ZMO0799***	***ABC*** **-** ***2 type transporter***	***2.35***	***3.64***	***2.17***
***Genetic/environmental/cellular Process/organismal systems***	***ZMO0800***	***ABC transporter***	***2.23***	***3.99***	***2.19***
Genetic/environmental/cellular process/organismal systems	*ZMO0801*	Secretion protein HlyD family protein	2.09	3.59	
***Genetic/environmental/cellular process/organismal systems***	***ZMO0965***	***Efflux pump membrane protein***	***2.08***	***5.02***	
Genetic/environmental/cellular process/organismal systems	*ZMO1253*	Cytochrome C biogenesis protein	2.76	2.45	
Genetic/environmental/cellular process/organismal systems	*ZMO1254*	Redoxin domain-containing protein	2.28	2.12	
Genetic/environmental/cellular process/organismal systems	*ZMO1527*	Acriflavin resistance protein	2.75	2.11	
Genetic/environmental/cellular process/organismal systems	*ZMO1528*	Acriflavin resistance protein	4.24	3.28	
Genetic/environmental/cellular process/organismal systems	*ZMO1530*	KpsF/GutQ family protein	3.34	2.13	
Genetic/environmental/cellular process/organismal systems	*ZMO1601*	Ribonuclease H	2.08	2.13	
Genetic/environmental/cellular process/organismal systems	*ZMO1727*	Aminotransferase	3.02	2.09	
Unassigned	*ZMO0020*	Hypothetical protein	3.08	2.14	2.36
Unassigned	*ZMO0021*	Hypothetical protein	3.11	2.89	2.38
Unassigned	*ZMO0073*	CBS domain-containing protein	5.98	4.13	
Unassigned	*ZMO0157*	d-isomer-specific 2-hydroxyacid dehydrogenase NAD-binding protein	2.28	2.22	
Unassigned	*ZMO0268*	Hypothetical protein	2.32	2.70	
Unassigned	*ZMO0270*	Hypothetical protein	2.41	2.91	
Unassigned	*ZMO0384*	Hypothetical protein		2.89	2.07
Unassigned	*ZMO0388*	Hypothetical protein		2.30	2.15
Unassigned	*ZMO0391*	Hypothetical protein		2.76	2.22
Unassigned	*ZMO0440*	Hypothetical protein	2.25	2.36	
Unassigned	*ZMO0683*	Csy2 family CRISPR-associated protein	2.55	2.47	
Unassigned	*ZMO0757*	TPR repeat-containing protein	2.02	2.92	
Unassigned	*ZMO0758*	Isochorismatase hydrolase	2.41	2.55	
Unassigned	*ZMO0763*	Tetratricopeptide domain-containing protein	2.48	2.01	
***Unassigned***	***ZMO0798***	***NodT family RND efflux system outer membranelipoprotein***	***2.27***	***4.58***	***2.54***
Unassigned	*ZMO0844*	Sporulation domain-containing protein	2.32	2.09	
Unassigned	*ZMO1334*	Hypothetical protein	4.11	2.96	
Unassigned	*ZMO1346*	Hypothetical protein	2.23	2.39	
Unassigned	*ZMO1380*	AraC family transcriptional regulator	5.17	2.60	2.21
Unassigned	*ZMO1386*	Hypothetical protein	3.44	2.39	
Unassigned	*ZMO1391*	Diacylglycerol kinase catalytic region	2.42	2.69	
Unassigned	*ZMO1399*	Fatty acid hydroxylase	3.94	2.98	
Unassigned	*ZMO1406*	Alpha/beta hydrolase	3.94	2.26	
Unassigned	*ZMO1437*	Lysine exporter protein	3.72	2.42	2.81
***Unassigned***	***ZMO1529***	***RND family efflux transporter subunit MFP***	***2.54***	***2.32***	
**Unassigned**	***ZMO1576***	**Short-chain dehydrogenase/reductase SDR**	**3.77**	**2.17**	
Unassigned	*ZMO1602*	Hypothetical protein	2.77	2.17	
**Unassigned**	***ZMO1696***	**Zinc-binding alcohol dehydrogenase**	**2.46**	**2.94**	
Unassigned	*ZMO1821*	Hypothetical protein	3.62	4.26	
Unassigned	*ZMO1880*	Hypothetical protein	2.58	2.90	
**Unassigned**	***ZMO1885***	**NADH:flavin oxidoreductase/NADH oxidase**	**11.37**	**3.33**	**2.67**
**Unassigned**	***ZMO1984***	**Aldo/keto reductase**	**3.87**	**2.06**	
	*ZZM4_0133*	Hypothetical protein	2.39	2.75	
	*pzmob1_p38*	Hypothetical protein	2.29	2.02	

^a^Expression levels with at least twofold or greater increase in response to phenolic aldehyde functional *p*-hydroxyphenyl group (H), guaiacyl group (G), and syringyl group (S), represented by 4-hydroxybenzaldehyde, vanillin, and syringaldehyde, respectively

^b^Bolded indicates reductase-related functional genes

^c^Bolded italic indicates transporter-related functional genes

The results show that the transcriptome response to syringaldehyde stress for *Z. mobilis* was significantly different from that to 4-hydroxybenzaldehyde and vanillin stress. The possible reasons may come from the following two aspects: (1) low molecular weight. The molecular weights of 4-hydroxybenzaldehyde, syringaldehyde, and vanillin were 122.1, 182.2, and 152.2, respectively. Similar to *S. cerevisiae* to low molecular weights phenolic compounds [1], the low molecular weight syringaldehyde gave the least toxicity for *Z. mobilis* ZM4. (2) Substituent position. The methoxyl substituent at *ortho* position increases the toxicity of vanillins [13], while the methoxyl and hydroxyl substituents at *meta* and *para* positions or vice versa do not change the toxicity [1]. Vanillin has one more methoxyl group and syringaldehyde has two more methoxyl groups on 4-hydroxybenzaldehyde. The configuration of the two methoxyl groups may generate space shelling for the toxic methoxyl substituent at *ortho* position, and then reduced the toxicity of syringaldehyde.

### Important reductase genes

**Fig. 5:**
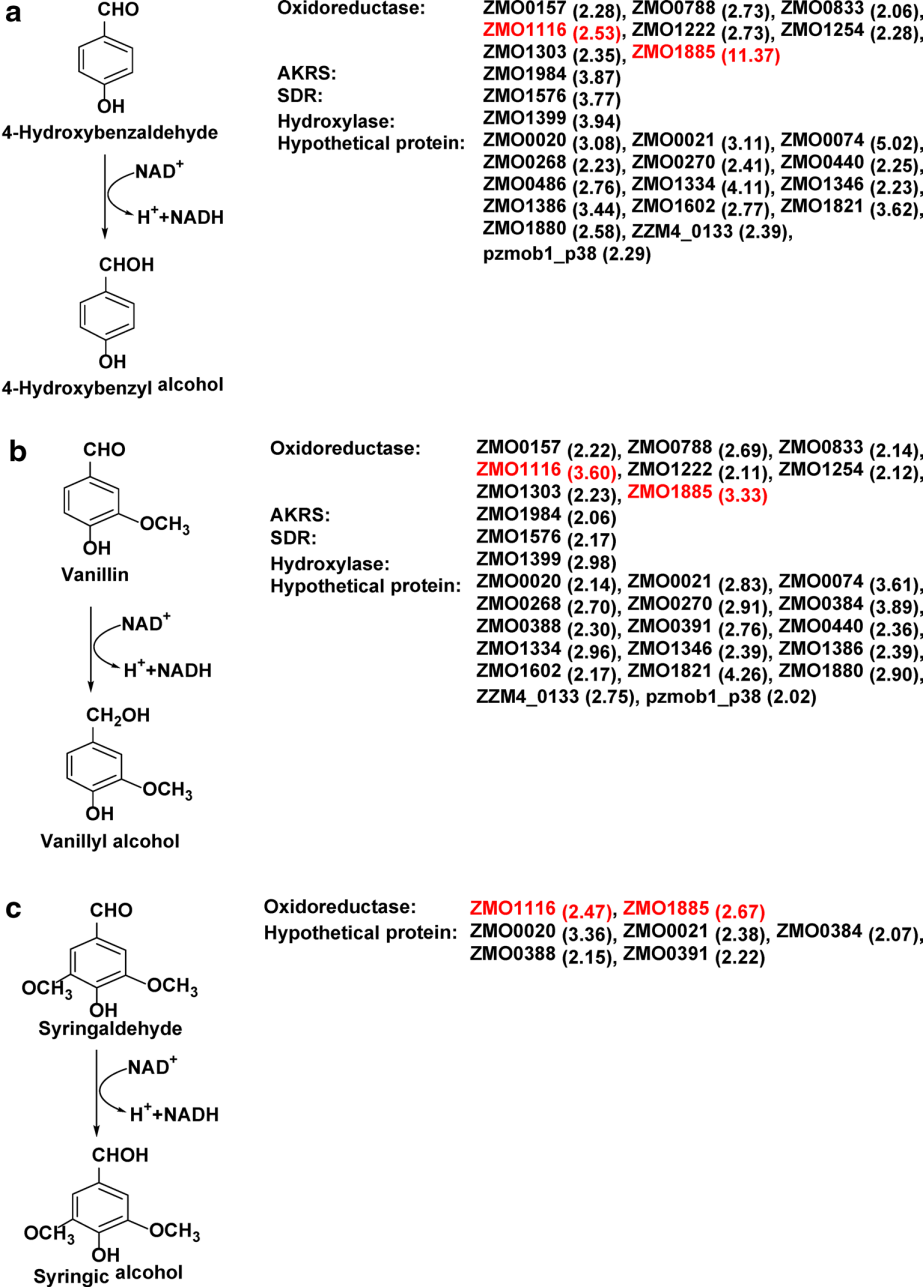
Conversion pathways of phenolic aldehydes. **a** Reduction of 4-hydroxybenzaldehyde, **b** 4-hydroxy-3-methoxybenzaldehyde (vanillin), and **c** syringaldehyde into the corresponding phenolic alcohols by candidate genes using NADH as a cofactor. Genes colored *red* indicate the most significant shared by all three phenolic aldehydes

Among many highly induced expressions, two reductase genes, *ZMO1116* encoding oxidoreductase and *ZMO1885* encoding NADH: flavin oxidoreductase/NADH oxidase, were outstanding in response to the challenge of all three phenolic aldehydes. *ZMO1116* was up-regulated expression with 2.53-, 2.47-, and 3.60-fold changes against 4-hydroxybenzaldehyde, vanillin, and syringaldehyde, respectively (Table 1), and *ZMO1885* was up-regulated by 11.37-, 2.67-, and 3.33-fold changes. Other genes, *ZMO0157* encoded d-isomer specific 2-hydroxyacid dehydrogenase NAD binding, *ZMO0788* encoded a gluconate 2-dehydrogenase, *ZMO0833* encoded UDP-*N*-acetylenolpyruvoylglucosamine reductase, *ZMO1222* encoded 3-oxoacyl-(acyl-carrier-protein) reductase, *ZMO1254* encoded redoxin domain protein, *ZMO1303* encoded a pyrroline-5-carboxylate reductase, *ZMO1399* encoded fatty acid hydroxylase, *ZMO1576* encoded short-chain dehydrogenase/reductase SDR, *ZMO1696* encoding zinc-binding alcohol dehydrogenase, *ZMO1984* encoded aldo/keto reductase, were also up-regulated over twofold change under at least two phenolic aldehyde inhibitors. *ZMO0020*, *ZMO0021*, *ZMO0074*, *ZMO0268*, *ZMO0270*, *ZMO0384*, *ZMO0388*, *ZMO0391*, *ZMO0440*, *ZMO0486*, *ZMO1334*, *ZMO1346*, *ZMO1386*, *ZMO1602*, *ZMO1821*, *ZMO1880*, *ZZM4_0133*, and *pzmob1_p38* encoded hypothetical proteins, also showed high levels of enhanced expressions in response to at least two phenolic aldehyde inhibitors. Figure 5 illustrates the degradation pathways of the three phenolic aldehydes in *Z. mobilis* ZM4 with the information of significantly differentially expressed genes.

### Significant transporter genes for detoxification

Efflux system of living cells is an efficient mechanism for detoxification of external toxic compounds and internal damaging intermediates. We found at least four transporter genes were significantly up-regulated in response to the challenge of phenolic aldehydes. Two genes *ZMO0799* and *ZMO0800* belonging to adenosine triphosphate (ATP)-binding cassette (ABC) superfamily showed consistently greater expressions to all the three phenolic aldehydes (Table 1). Other two transporter genes, *ZMO0282* and *ZMO0798* belonging to resistance-nodulation-cell division (RND) superfamily, showed up to sevenfold increased expression to the phenolic challenge. One gene *ZMO1288* encoding a major facilitator superfamily (MFS) transporter protein was down-regulated more than twofold change. To limit the intracellular concentration of toxic inhibitors, Gram-negative bacteria can diminish its entry by developing a barrier of low permeability [14]. This non-specific phenomenon is also existing in *Z. mobilis* ZM4, such as the down-regulation of *ZMO1288* encoding a major facilitator superfamily (MFS) transporter protein.

### Overexpression and tolerance evaluation of the important genes in *Z. mobilis* ZM4

Genes with strong response to phenolic aldehydes on the transcriptional level were screened and overexpressed in *Z. mobilis* ZM4 to confirm their functions. Four genes, *ZMO1116*, *ZMO1288*, *ZMO1696*, and *ZMO1885*, were selected, including (1) significantly up-regulated genes: *ZMO1116* encoding an oxidoreductase with up-regulation by 2.53-, 2.47-, and 3.60-fold change, and *ZMO1885* encoding a NADH:flavin oxidoreductase/NADH oxidase with 11.37-, 2.67-, and 3.33-fold change under the stress of 4-hydroxybenzaldehyde, syringaldehyde, and vanillin, respectively (data shown in Table 1); (2) important genes on the degradation pathway with relatively high up-regulation: *ZMO1696* encoding zinc-binding alcohol dehydrogenase with up-regulation by 2.46-, 1.52-, and 2.94-fold change under the same stress (data shown in Table 1); (3) significantly down-regulated gene: *ZMO1288* encoding a major facilitator superfamily (MFS) transporter protein with down-regulation by 4.07-, 4.18-, and 3.44-fold change under the above same stress (see Additional file 4). The selection of *ZMO1288* for overexpression is to validate whether the down-regulation of *ZMO1288* contributed to the resistance of phenolic aldehyde inhibitors.

**Fig. 6:**
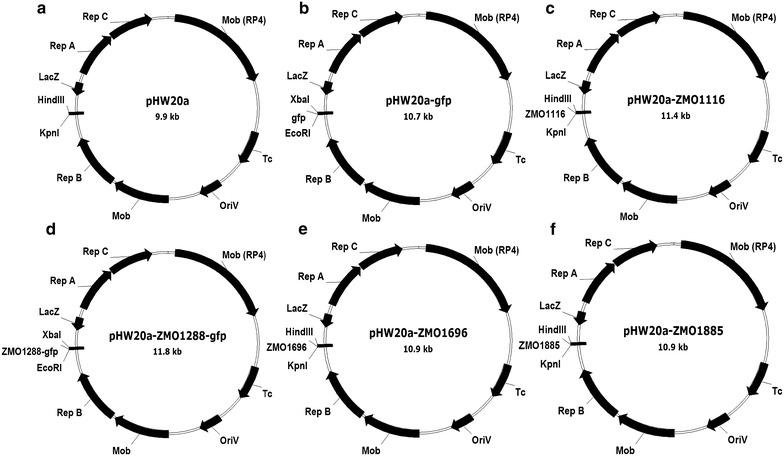
Construction of the plasmids for expression of functional genes with strong response to phenolic aldehydes. **a** pHW20a. **b** pHW20a-*gfp*. **c** pHW20a-*ZMO1116*. **d** pHW20a-*ZMO1288*-*gfp*. **e** pHW20a-*ZMO1696*. **f** pHW20a-*ZMO1885*

Four pHW20a plasmids harboring *ZMO1116*, *ZMO1288*, *ZMO1696*, or *ZMO1885* (Fig. 6) were introduced into *Z. mobilis* ZM4 to generate the recombinants according to the protocol in Dong et al. [15]. The shuttle expression vector pHW20a, as well as pHW20a-*gfp* with *gfp* and a short linker were used as the two controls. The successful expression of *ZMO1116*, *ZMO1696*, and *ZMO1885* were confirmed by vanillin degradation by the recombinants at the rate of 0.112, 0.125, and 0.023 μmol per minute, respectively. The expression of the transporter gene *ZMO1288* was confirmed by the fluorescence intensity of 10,340 at 507 nm for the recombinant with the fused *gfp*, comparing to 9497 of the control containing only *gfp* reporter gene. The recombinants were cultured in RM medium separately amended with 5 mM of 4-hydroxybenzaldehyde, syringaldehyde, or vanillin. The cell growth, glucose consumption rate, ethanol productivity, and phenolic aldehyde inhibitors conversion rate were measured as shown in Table 2. Under the stress of 4-hydroxybenzaldehyde, the overexpression of *ZMO1116* increased the cell growth, glucose consumption, ethanol productivity, and 4-hydroxybenzaldehyde conversion by 3.92, 23.39, 13.64, and 10.92 %, respectively. Overexpression of *ZMO1288* led to the increases of the above parameters by 10.42, 60.84, 50.00, and 86.84 %, respectively. Overexpression of *ZMO1696* led to the increases of the above parameters by 3.92, 20.00, 13.64, and 133.48 %, respectively. Overexpression of *ZMO1885* led to the increases of the above parameters by 5.88, 31.61, 18.18, and 52.11 %, respectively. On the other hand, the expression of all the genes on cell growth increased a little. Under the stress of syringaldehyde, the overexpression of *ZMO1116*, *ZMO1288*, *ZMO1696*, and *ZMO1885* increased syringaldehyde conversion by 18.40, 51.87, 20.43, and 30.47 %, respectively, but the cell growth, glucose consumption, and ethanol productivity only showed limited change. Under the stress of vanillin, the overexpression of *ZMO1116* increased the cell growth, glucose consumption, ethanol productivity, and vanillin conversion by 40.68, 16.32, 11.76, and 19.49 %, respectively. Overexpression of *ZMO1288* led to the increases of 45.90, 19.10, 14.29, and 210.23 %, respectively. Overexpression of *ZMO1696* led to the increases of 8.47, 20.52, 7.84, and 193.93 %, respectively. Overexpression of *ZMO1885* led to the increases of 16.95, 8.16, 13.73, and 97.43 %, respectively.

**Table 2:** Evaluation of selective *Z. mobilis* recombinants with genes showing differential expressions in genome expression analysis

Inhibitors	Recombinants	Cell growth (OD_600_)	Glucose consumption (g/L)	Ethanol productivity (g/L/h)	Inhibitors conversion (%)
4-Hydroxybenzaldehyde (H group)	*ZM4::pHW20a*	0.51 ± 0.01	5.60 ± 0.99	0.22 ± 0.00	6.87 ± 0.25
	*ZM4::pHW20a*-*ZMO1116*	0.53 ± 0.04	6.91 ± 0.38	0.25 ± 0.00	7.62 ± 0.68
	*ZM4::pHW20a*-*ZMO1696*	0.53 ± 0.01	6.72 ± 1.32	0.25 ± 0.00	16.04 ± 0.86
	*ZM4::pHW20a*-*ZMO1885*	0.54 ± 0.01	7.37 ± 0.05	0.26 ± 0.00	10.45 ± 0.94
	ZM4:pHW20a-*gfp*	0.48 ± 0.01	6.64 ± 1.58	0.16 ± 0.18	7.52 ± 0.48
	ZM4:pHW20a-*ZMO1288*-*gfp*	0.53 ± 0.04	10.68 ± 0.69	0.24 ± 0.00	14.05 ± 1.37
Syringaldehyde (S group)	*ZM4::pHW20a*	1.26 ± 0.03	16.58 ± 0.39	0.58 ± 0.01	8.37 ± 0.67
	*ZM4::pHW20a*-*ZMO1116*	1.29 ± 0.02	16.98 ± 0.16	0.60 ± 0.01	9.91 ± 0.08
	*ZM4::pHW20a*-*ZMO1696*	1.33 ± 0.00	16.99 ± 0.06	0.57 ± 0.03	10.08 ± 1.09
	*ZM4::pHW20a*-*ZMO1885*	1.36 ± 0.00	17.02 ± 0.09	0.61 ± 0.02	10.92 ± 0.40
	ZM4:pHW20a-*gfp*	1.29 ± 0.01	16.88 ± 0.02	0.60 ± 0.00	7.23 ± 0.38
	ZM4:pHW20a-*ZMO1288*-*gfp*	1.30 ± 0.00	16.76 ± 0.05	0.60 ± 0.00	10.98 ± 0.83
Vanillin (G group)	*ZM4::pHW20a*	0.59 ± 0.03	12.62 ± 0.33	0.51 ± 0.24	5.44 ± 0.71
	*ZM4::pHW20a*-*ZMO1116*	0.83 ± 0.07	14.68 ± 0.05	0.57 ± 0.00	6.50 ± 0.87
	*ZM4::pHW20a*-*ZMO1696*	0.64 ± 0.01	15.21 ± 0.37	0.55 ± 0.01	15.99 ± 1.13
	*ZM4::pHW20a*-*ZMO1885*	0.69 ± 0.01	13.65 ± 0.20	0.58 ± 0.01	10.74 ± 0.13
	*ZM4::pHW20a*-*gfp*	0.61 ± 0.03	11.52 ± 1.58	0.42 ± 0.02	5.18 ± 0.59
	*ZM4::pHW20a*-*ZMO1288*-*gfp*	0.89 ± 0.05	13.72 ± 0.25	0.48 ± 0.01	16.07 ± 0.07

The low toxicity of syringaldehyde to *Z. mobilis* led to the limited inhibitory influence on cell growth and ethanol productivity, so the reduced level of syringaldehyde by the overexpression of the key genes also did not increase the cell growth and ethanol productivity. But the obvious improvements of cell growth and ethanol fermentation were obtained for the more toxic 4-hydroxybenzaldehyde and vanillin for inhibitor conversion.

Overexpression of the reductase genes *ZMO1116*, *ZMO1696*, and *ZMO1885* in *Z. mobilis* ZM4 significantly improved the tolerance to phenolic aldehydes, indicating the importance of reductases for phenolic aldehydes conversion. Müller et al. [16] found the reduction activity of NAD(P)H-dependent reductase encoded by *ZMO1885* from *Z. mobilis* on the carbon double bond, and this study also verified the reduction activity of phenolic aldehydes into phenolic alcohols. Overexpression of *ZMO1288* encoding a major facilitator superfamily (MFS) protein contributed to the highest inhibitor conversion, suggesting that the increase of toxin transport into intracellular space by enhancing *ZMO1288* activity did not harm the intracellular activity. Instead, the enhanced transport of inhibitors to intracellular space facilitated the fast degradation and reduction of the toxic phenolic aldehydes.

## Discussion

In this study, our transcriptome analysis using microarray revealed the first insight into genomic response of *Z. mobilis* ZM4 to the three groups of phenolic aldehyde inhibitors derived from lignocellulose pretreatment. We identified 272 up-regulated genes, including 36 gene clusters, against phenolic aldehydes as the potential candidate tolerance genes. We also identified significantly repressed genes and gene clusters to the phenolic aldehydes and provided their physical locations in the genome map. Furthermore, we illustrated mechanisms of bacterial tolerance and detoxification of these phenolic aldehydes through a group of genes encoding varied reductases and transporters. New knowledge obtained from this research aids understanding of the bacterial tolerance and the development of strong inhibitor tolerant fermenting strains.

Phenolic aldehydes are among the most toxic inhibitory compounds for microbial growth and fermentation. The toxicity is often thought caused by aromatic ring and the mechanism of inhibition is not clear. It is generally accepted that these phenolic compounds are classified into three groups of *p*-hydroxyphenyl group (H), guaiacyl group (G), and syringyl group (S) based on their methoxyl groups [1]. We used three phenolic aldehydes of 4-hydroxybenzaldehyde, vanillin, and syringaldehyde to represent each of these three different phenolic groups. We observed in the *Z. mobilis* ZM4 cultures, phenolic aldehydes concentrations were reduced and the corresponding alcohols were increased. As a result, when the phenolic aldehydes were reduced to certain lower levels, *Z. mobilis* ZM4 cells were able to accelerate the rate of glucose consumption and cell growth and eventually complete the fermentation. As demonstrated by our GC–MS analysis, 4-hydroxybenzaldehyde was converted into 4-hydroxybenzyl alcohol, and vanillin into vanillyl alcohol, and syringaldehyde into syringic alcohol. In these corresponding alcohols, a typical aromatic ring still existed but it apparently did not cause inhibition to the cell growth and metabolism of *Z. mobilis* ZM4. Functional group of aldehyde is apparently the toxic component of these phenolic aldehydes, regardless of their classification of phenolic group H, G, or S, based on the mass balance of phenolic aldehydes and its alcohol or acid derivatives, as well as the almost constant total phenolic concentrations. In response to the challenge of these phenolic aldehydes, the gene expression of *ZMO0157*, *ZMO0788*, *ZMO0833*, *ZMO1116*, *ZMO1222*, *ZMO1254*, *ZMO1303*, *ZMO1399*, *ZMO1576*, *ZMO1696*, *ZMO1885*, and *ZMO1984* was significantly up-regulated under the stress of at least two phenolic aldehydes. These genes were found primarily encoding oxidoreductase, alcohol dehydrogenase, oxidoreductase/NADH oxidase, aldo/keto reductase, and a member of short-chain dehydrogenase/reductase (SDR), respectively. Our results suggest these genes are commonly involved in the conversion of phenolic aldehydes into the corresponding phenolic alcohols. However, since the complexity of the compound structure differs, the fine toned mechanism of the conversion pathway can be different. For example, syringaldehyde is more stable due to its complex structure and may require additional conversion pathways. It indicated that reduction and transport contributed to the conversion of the phenolic aldehydes into the corresponding phenolic alcohols.

Similarly, furfural and HMF were conventionally called furan inhibitors that hindered studies on microbial tolerance to these common biomass fermentation inhibitors. He et al. [9, 11] has found 127 genes and 433 genes differentially expressed in *Z. mobilis* in response to ethanol and furfural by DNA microarray, respectively. Metabolic conversion pathways of furfural and HMF into furanmethanol and furan-2,5-dimethanol by tolerant yeast *Saccharomyces cerevisiae* NRRL Y-50049 were presented and the toxic functional group of aldehyde, not furan group, was clarified [17–19]. It appeared there might be an aldehyde reductase gene family exist [20, 21]. In this study, we observed multiple enzymes were involved in the conversion of phenolic aldehydes into corresponding alcohols that is consistent with previous reported. The overexpression of oxidoreductase (*ZMO1116*), zinc-binding alcohol dehydrogenase (*ZMO1696*), and NADH:flavin oxidoreductase/NADH oxidase (*ZMO1885*) with significantly enhanced conversion of phenolic aldehydes into phenolic alcohols gave further supports the multiple gene involvement in microbe tolerance to aldehyde inhibitors.

In our study, we also observed expression levels of at least eight transporter genes were significantly increased in response to varied phenolic aldehydes, including *ZMO0143*, *ZMO0799*, and *ZMO0800* from ATP-binding cassette (ABC) superfamily; *ZMO0282*, *ZMO0798*, and *ZMO1529* from RND (resistance-nodulation-cell division) superfamily; and *ZMO0283* and *ZMO0965* from efflux system proteins. In yeast, numerous transporter genes were identified to be involved in response to aldehyde including ABC transporter genes and other drug and toxin transporter genes [22]. Efflux pumps from RND family are considered one of the most efficient mechanisms for tolerance to toxin in Gram-negative bacteria [23]. Results of this study in *Z. mobilis* ZM4 in response to phenolic aldehyde inhibitors concur with the conclusion obtained from *S. cerevisiae*.

Our comprehensive analysis of the genome expression in *Z. mobilis* ZM4 provided the first important gene atlas for significantly enhanced and repressed genes at the genomic level. This illustrated distribution of potential candidate tolerance genes and gene clusters at the genomic landscape can serve as a valuable resource for genetic engineering efforts in genomic manipulation of *Z. mobilis* ZM4 for the continued tolerant strain development. In general, enhanced expressed genes under the inhibitor challenge pressure are considered as tolerance genes. However, this has to be in the context of consistent dynamics over a time course. In this study, we took merely a snapshot in the mid of the logarithm growth phase, thus, a more conserved approach should be taken in selection of candidate tolerance genes. On the other hand, it needs to point out that repressed genes are also important, particularly for those recovered from the early repression. Such genes often contribute to adaptation, redirecting gene interactions, and rewired networks for survival and tolerance development as demonstrated in *S. cerevisiae* [24, 25].

## Conclusions

The ethanologenic bacterium *Z. mobilis* ZM4 degrades phenolic aldehydes into their corresponding phenolic alcohols forms, and the toxicity of phenolic aldehydes to cell growth and ethanol fermentation is caused by the functional group of phenolic aldehyde rather than the phenolic aromatic ring. Among 272 up-regulated genes, including 36 gene clusters, reductases are the potential candidate tolerance genes for the reduction of phenolic aldehydes. Tolerant genes identified in this study will serve as valuable resources for robust strain development for future biorefinery applications.

## Methods

### Bacterial strain, plasmid, and reagents

*Zymomonas mobilis* ZM4 (ATCC 31821) was purchased from American Type Culture Collection (Manassas, VA, USA). Shuttle vector pHW20a was used for construction of *Z. mobilis* recombinants [15]. Yeast extract was purchased from Oxoid (Hampshire, UK). Tetracycline (Tc) and nalidixic acid were from Sigma-Aldrich, St. Louis, MO, USA. 4-Hydroxybenzaldehyde, syringaldehyde, and vanillin were from Sangon Biotech Co., Shanghai, China. All other analytical grade chemicals were from Sinopharm Chemical Reagents, Shanghai, China.

### Cell growth conditions

Cells of *Z. mobilis* ZM4 were cultured and maintained in RM medium containing 20 g/L glucose, 2 g/L KH_2_PO_4_, and 10 g/L yeast extract. The initial culture was incubated at 30 °C without agitation. RM medium was amended separately by adding 5 mM each of 4-hydroxybenzaldehyde, syringaldehyde, or vanillin for testing the inhibitor tolerance. A non-phenolic aldehyde-treated culture was served as control. Samples were taken at a 4 h interval till 36 h. For DNA microarray, 10 mL culture was transferred into 100 mL fresh RM medium when the culture was 2.0 at the optical density at 600 nm. Cells were harvested at 4 h for RNA extraction, DNA microarray, and quantitative real-time PCR (qRT-PCR).

### DNA microarray

Total RNA of *Z. mobilis* ZM4 cells was extracted using a Trizol reagent kit from Invitrogen (Carlsbad, CA, USA). Purity and concentrations of the RNA samples were measured by a ratio of OD_260_/_280_ readings using a NanoDrop ND-1000 spectrophotometer. DNA microarray was performed by CapitalBio Co., Beijing, China. cDNA fragments were hybridized with the probes and labeled with Cy3-dCTP fluorescent dye [26]. Data were analyzed using GeneSpring V12 Agilent Technologies (Santa Clara, CA, USA) with proper normalization and quality control procedures. Raw data were transformed into algorism phase based on Log_2_ and statistical analysis was carried out using software CLUSTER 3.0 [27]. Significance of differential expression levels compared with the control was examined using False Discovery Rate (FDR) test (*p* < 0.05) [28] and analysis of variance (ANOVA). A threshold of fluorescence intensity with more or less than twofold changes was regarded as significantly differentially expressed genes. Finally, a hierarchical clustering analysis was performed using Java Treeview (Stanford University, Stanford, CA, USA).

### qRT-PCR assays

To confirm expression data from microarray, the quantitative mRNA expressions were performed using qRT-PCR on a CFX96TM Real-Time System with C10000TM Thermal Cycler from Bio-Rad Laboratories (Hercules, CA, USA). Primers for qRT-PCR of the selected genes are shown in Additional file 2. The first strand of cDNA was synthesized using a cDNA synthesis kit (Torobo Co., Osaka, Japan). PCR amplification was prepared using an SYBR Green Real-time PCR Master Mix. PCR amplification profile used was follows: 94 °C for 5 min, then 35 cycles at 94 °C for 2 min and 62 °C for 30 s, and 72 °C for 30 s. 16S rRNA gene *ZMOr003* was used as an internal control for data acquisition and normalization.

### Construction of *Z. mobilis* recombinants

Genomic DNA of *Z. mobilis* ZM4 was extracted using Omega-Biotek Bacterial DNA kit (Norcross, GA, USA). Primers for amplification of *ZMO1116*, *ZMO1288*, *ZMO1696*, and *ZMO1885* genes were listed in Additional file 3. Construction of the plasmids harboring *ZMO1116*, *ZMO1288*, *ZMO1696*, and *ZMO1885* genes is illustrated in Fig. 6. The transporter gene *ZMO1288* was fused with a reporter gene *gfp* which was amplified from the plasmid pMG36E [29], with a short linker encoding seven glycines at the C-terminal of *gfp* between *gfp* and *ZMO1288* for confirmation of its expression. The plasmids were introduced into *E. coli* S17-1 λπ, and then into *Z. mobilis* ZM4 through biparental transconjugation method [15].

### Evaluation of *Z. mobilis* ZM4 recombinants

The oxidoreductase activity of the recombinants was evaluated in 200 μL of reaction mixture containing 0.1 M potassium phosphate buffer (pH 7.5), 0.329 mM vanillin, 2.25 mM NADH, and 20 μL cell-free extract [30] at 30 °C for 30 min, and then stopped by addition of 10 μL of 3 M trichloroacetic acid solution and measured vanillin reduction by HPLC. One oxidoreductase unit (U) was defined as the amount of the crude enzyme required to reduce 1 μmol of vanillin per min at pH 7.5 and 25 °C. The expression of transporter gene *ZMO1288* fused with *gfp* gene was detected by monitoring fluorescence using Synergy H1 Hybrid Microplate Reader at 507 nm (Biotek Instruments, Winooski, VT, USA). Fermentation performance of the newly generated *Z. mobilis* recombinants was evaluated as described above using RM medium separately amended with 5 mM each of 4-hydroxybenzaldehyde, syringaldehyde, or vanillin. Samples were periodically taken from the culture broth and supernatant obtained by centrifuging at 12,000*g* for 5 min and filtering through 0.22-μm filters.

### HPLC and GC–MS analysis

Glucose and ethanol were analyzed using HPLC equipped with a refractive index detector RID-10A (Shimadzu, Kyoto, Japan) with an Aminex HPX-87H column (Bio-rad, Hercules, CA, USA) at 65 °C using a mobile phase of 5 mM H_2_SO_4_ at a rate of 0.6 mL/min. 4-Hydroxybenzaldehyde, syringaldehyde, and vanillin were analyzed using reverse-phase HPLC (SPD-20A, Shimadzu, Kyoto, Japan) equipped with a YMC-Pack ODS-A column (Tokyo, Japan) at 35 °C. 4-Hydroxybenzaldehyde and syringaldehyde were detected at 270 nm using a mobile phase of 30 % acetonitrile solution at a rate of 1.0 mL/min, and vanillin was detected at 320 nm using 50 % acetonitrile solution at a rate of 0.8 mL/min.

The biodegradation intermediates of phenolic aldehydes by *Z. mobilis* ZM4 was identified by GC–MS. Samples were taken at 4 h intervals after inoculation and concentrated by rotary evaporator with vacuum system, then dissolved in ethyl acetate and acetonitrile solution (2:1, v/v) and silylated with NO-bis-trimethylsilyl trifluoro-acetamide according to [31, 32]. The treated samples were analyzed using Agilent 6890 GC–MS fitted with an HP-5 MS column (30 m × 0.25 mm × 0.25 μm) (Agilent Technologies, Santa Clara, CA, USA) from 80 °C (held for 4 min) to 280 °C at 8 °C/min. 1 μL sample was injected and detected under splitless condition.

## Additional files

**Additional file 1.** Degradation products of phenolic aldehydes for *Z. mobilis* ZM4 by GC–MS.
**Additional file 2.** Primers for qRT-PCR of the genes.
**Additional file 3.** Primers for the construction of the recombinant *Z. mobilis* ZM4.
**Additional file 4.** Differentially expressed genes (>2-fold change) under the stress of the phenolic aldehydes in *Z. mobilis* ZM4.

## Abbreviations

ATP: adenosine triphosphate
ABC: adenosine triphosphate (ATP)-binding cassette
cDNA: complementary DNA
dCTP: 2′-deoxycytidine 5′-triphosphate
FDR: False Discovery Rate
G: guaiacyl group
GC–MS: gas chromatography–mass spectrometer
gfp: green fluorescent protein
H: *p*-hydroxyphenyl group
5-HMF: 5-hydroxymethylfurfural
HPLC: high-performance liquid chromatography
MFS: major facilitator superfamily
NADH: nicotinamide adenine dinucleotide
OD: optical density
qRT-PCR: quantitative real-time polymerase chain reaction
RM: rich medium
RND: resistance-nodulation-cell division
S: syringyl group
ZMO: *Zymomonas mobilis* open reading frame
SDR: short-chain dehydrogenase/reductase
