# Supplementary material for: Transcriptome analysis of Zymomonas mobilis ZM4 reveals mechanisms of tolerance and detoxification of phenolic aldehyde inhibitors from lignocellulose pretreatment
Source: Biotechnol Biofuels. 2015 Sep 22;8:153. doi: 10.1186/s13068-015-0333-9 (PMC4578398; doi:10.1186/s13068-015-0333-9)
Supplement: Supplementary file 1 — Additional file 1. Degradation products of phenolic aldehydes for Z. mobilis ZM4 by GC–MS. [file 13068_2015_333_MOESM1_ESM.docx]

**Additional file 1 Degradation products of phenolic aldehydes for *Z. mobilis* ZM4 by GC-MS.**

| Substrate | Metabolic products | Molecular formula | MW | RT (min) |
| --- | --- | --- | --- | --- |
| 4-Hydroxybenzaldehyde | 4-Hydroxybenzaldehyde | C_7_H_6_O_2_ | 122.04 | 12.13 |
|  | 4-Hydroxybenzyl alcohol | C_13_H_24_O_2_Si_2_ | 268.13 | 19.46 |
| Syringaldehyde | Syringaldehyde | C_9_H_10_O_4_ | 182.06 | 19.55 |
|  | Syringic alcohol | C_9_H_12_O_4_ | 184.07 | 20.40 |
| Vanillin | Vanillin | C_8_H_8_O_3_ | 152.05 | 14.14 |
|  | Vanillyl alcohol | C_8_H_10_O_3_ | 154.06 | 15.23 |
