# Supplementary material for: Transcriptome analysis of Zymomonas mobilis ZM4 reveals mechanisms of tolerance and detoxification of phenolic aldehyde inhibitors from lignocellulose pretreatment
Source: Biotechnol Biofuels. 2015 Sep 22;8:153. doi: 10.1186/s13068-015-0333-9 (PMC4578398; doi:10.1186/s13068-015-0333-9)
Supplement: Supplementary file 2 — Additional file 2. Primers for qRT-PCR of the genes. [file 13068_2015_333_MOESM2_ESM.docx]

**Additional file 2 Primers for qRT-PCR of the genes.**

| **Gene** | **Primer ID** | **Forward primer (5'-3')** |
| --- | --- | --- |
| ZMO0152 | ZMO0152F | TCTGACCCCGAAAGATCGCA |
|  | ZMO0152R | GATGACGTCTTCGACGCGCT |
| ZMO0177 | ZMO0177F | TCGGTTACACCGACGAACCC |
|  | ZMO0177R | ACAACGCGAGCCAGCTTACC |
| ZMO0179 | ZMO0179F | AGAATTCCGCCATATTGCGG |
|  | ZMO0179R | AGGCACTGACATCCAAGGCC |
| ZMO0367 | ZMO0367F | TTCGACGGTTCTGGCAGCTT |
|  | ZMO0367R | TTCGTCACGAACAGCGTTGG |
| ZMO0368 | ZMO0368F | GTCACGACCATTGCCAAGGG |
|  | ZMO0368R | TCATCACCAGGTTCGCCAGC |
| ZMO0369 | ZMO0369F | AACGCATTATTTCTGGCCCG |
|  | ZMO0369R | CAAGCTGAACGGAACGCCTT |
| ZMO0387 | ZMO0387F | GTTTCCGCCGCGTTTCTATC |
|  | ZMO0387R | CAAGCTGAACGGAACGCCTT |
| ZMO0543 | ZMO0543F | GGCGTGAAGGCGCTGATAAA |
|  | ZMO0543R | TCCGGTGAGCAAACAAAGGC |
| ZMO0544 | ZMO0544F | GCGGTATTGGTATCGCACCG |
|  | ZMO0544R | CTTCCCCAGCGATATCCGGT |
| ZMO0567 | ZMO0567F | TCGGATGAAGAGAATGCCGC |
|  | ZMO0567R | CGCAGAAACACCGGCAATAA |
| ZMO0569 | ZMO0569F | ACCCTTAAACGGCTGCAACG |
|  | ZMO0569R | GCCCCAAACCAGCAAGGTTA |
| ZMO0997 | ZMO0997F | GGTATCACCCCGACGTCAGC |
|  | ZMO0997R | GTGCCGTGATTTTTGCGACA |
| ZMO1237 | ZMO1237F | TTTTCACGGGACATCAGGCC |
|  | ZMO1237R | TCCGGCATTTCTTTTCCAGC |
| ZMO1307 | ZMO1307F | ATCGGCATGATTGGCAAAGC |
|  | ZMO1307R | ATGAGATAAGCCGCACCCCC |
| ZMO1360 | ZMO1360F | CCCAACCCTGATCGAATGCT |
|  | ZMO1360R | ACAGGCTTACGGCTGTTGGC |
| ZMO1478 | ZMO1478F | CTGGCGATCATAAACGCACG |
|  | ZMO1478R | CGATGGAGGCTTCGGTTTCA |
| ZMO1496 | ZMO1496F | TGTTACCCGGTTGGTTCGGT |
|  | ZMO1496R | TAGTCGTGCGGAAAAACGGC |
| ZMO1596 | ZMO1596F | AAAGAAGGCGCAGAAGCCAC |
|  | ZMO1596R | TCAGCAAGAAGCGGCACATC |
| ZMO1608 | ZMO1608F | ACCACGATTGCTGACCTCGC |
|  | ZMO1608R | TCTTCGATGCGCATCAGCTG |
| ZMO1963 | ZMO1963F | CGCTTGGCTGGTTCTTCAGG |
|  | ZMO1963R | ATGTTGCCTCATTAGCGCCG |
