# Supplementary material for: Transcriptome analysis of Zymomonas mobilis ZM4 reveals mechanisms of tolerance and detoxification of phenolic aldehyde inhibitors from lignocellulose pretreatment
Source: Biotechnol Biofuels. 2015 Sep 22;8:153. doi: 10.1186/s13068-015-0333-9 (PMC4578398; doi:10.1186/s13068-015-0333-9)
Supplement: Supplementary file 3 — Additional file 3. Primers for the construction of the recombinant Z. mobilis ZM4. [file 13068_2015_333_MOESM3_ESM.docx]

**Additional file 3** **Primers for the construction of the recombinant *Z. mobilis* ZM4.**

| **Gene** | **Primer ID** | **Primer Sequence (5'-3')** |
| --- | --- | --- |
| *ZMO1116* | *ZMO1116*-F | CCCAAGCTTATGGCGCAAAATAAAATGCTG |
|  | *ZMO1116*-R | CGGGGTACCTCAGGCAAAAACAGCTTTCTTTT |
| *ZMO1288* | *ZMO1288*-P1 | GCTCTAGATGGAAAATGCGAGGAACGG |
|  | *ZMO1288*-P2 | ACCACCACCACCACCACCACCAACAAGCGCATCCGGCATT |
|  | *gfp*-P3 | TCAAAAAAAATGGCCGTGCAGGTGGTGGTGGTGGTGGTG |
|  | *gfp*-P4 | GGAATTCTTATTTGTATAGTTCATCCATGCCATG |
| *ZMO1696* | *ZMO1696*-F | CCCAAGCTTATGCGCGCCATAGGTTATC |
|  | *ZMO1696*-R | CGGGGTACCTTAGAAGCCTTCTAAGACGATTTTA |
| *ZMO1885* | *ZMO1885*-F | CCCAAGCTTATGCCTAGCTTGTTTGATCCCA |
|  | *ZMO1885*-R | CGGGGTACCTCAATCCCCAAGCAAAGGATAA |

Underline indicated the digestion sites.
