# Supplementary material for: Transcriptome analysis of Zymomonas mobilis ZM4 reveals mechanisms of tolerance and detoxification of phenolic aldehyde inhibitors from lignocellulose pretreatment
Source: Biotechnol Biofuels. 2015 Sep 22;8:153. doi: 10.1186/s13068-015-0333-9 (PMC4578398; doi:10.1186/s13068-015-0333-9)
Supplement: Supplementary file 4 — Additional file 4. Differentially expressed genes (>2-fold change) under the stress of the phenolic aldehydes in Z. mobilis ZM4. [file 13068_2015_333_MOESM4_ESM.docx]

**Additional file 4** **Differentially expressed genes (> 2-fold change) under the stress of the phenolic aldehydes in *Z. mobilis* ZM4.**

| **Catgory** | **Locus** | **Product** | **Up-regulated**  **fold-change** | | | **Down-regulated**  **fold-change** | | |
| --- | --- | --- | --- | --- | --- | --- | --- | --- |
|  |  |  | **H** | **G** | **S** | **H** | **G** | **S** |
| Amino Acid Metabolisms | ZMO0200 | Anthranilate phosphoribosyltransferase | 2.88 | 2.38 |  |  |  |  |
| Amino Acid Metabolisms | ZMO1303 | Pyrroline-5-carboxylate reductase | 2.35 | 2.23 |  |  |  |  |
| Amino Acid Metabolisms | ZMO1499 | Phosphoribosyl-ATP diphosphatase | 3.46 | 3.91 | 2.17 |  |  |  |
| Amino Acid Metabolisms | ZMO1500 | Imidazole glycerol phosphate synthase subunitHisF | 2.92 | 2.93 |  |  |  |  |
| Amino Acid Metabolisms | ZMO1501 | 1-(5-phosphoribosyl)-5-[(5-phosphoribosylamino) methylideneamino]imidazole-4-carboxamide isomerase | 2.30 | 2.10 |  |  |  |  |
| Amino Acid Metabolisms | ZMO1502 | Imidazole glycerol phosphate synthase subunitHisH | 2.14 | 2.25 |  |  |  |  |
| Biosynthesis of Other Secondary Metabolites | ZMO0456 | Ferredoxin |  |  |  | 3.87 | 2.18 |  |
| Carbohydrate Metabolism | ZMO0493 | Glutamine synthetase, type I | 2.80 | 3.21 | 2.52 |  |  |  |
| Carbohydrate Metabolism | ZMO0759 | Hydroxyacylglutathione hydrolase | 2.66 | 2.66 |  |  |  |  |
| Carbohydrate Metabolism | ZMO0788 | Gluconate 2-dehydrogenase | 2.73 | 2.69 |  |  |  |  |
| Carbohydrate Metabolism | ZMO0833 | UDP-N-acetylenolpyruvoylglucosamine reductase | 2.06 | 2.14 |  |  |  |  |
| Carbohydrate Metabolism | ZMO0903 | 2-isopropylmalate synthase |  |  |  | 5.26 | 4.89 |  |
| Carbohydrate Metabolism | ZMO1237 | D-isomer specific 2-hydroxyacid dehydrogenase NAD-binding protein |  |  |  | 4.85 | 2.39 | 4.66 |
| Energy Metabolism | ZMO0003 | Adenylylsulfate kinase | 2.40 | 2.01 |  |  |  |  |
| Energy Metabolism | ZMO0004 | Sulfate adenylyltransferase subunit 1 | 3.39 | 2.31 |  |  |  |  |
| Energy Metabolism | ZMO0005 | Sulfate adenylyltransferase subunit 2 | 3.09 | 2.13 |  |  |  |  |
| Energy Metabolism | ZMO1116 | Oxidoreductase | 2.53 | 3.60 | 2.47 |  |  |  |
| Genetic Information Processing/Environmental Information Processing/Cellular Process/Organismal Systems | ZMO0037 | PTS IIA-like nitrogen-regulatory protein PtsN |  |  |  |  | 2.41 | 2.07 |
| Genetic Information Processing/Environmental Information Processing/Cellular Process/Organismal Systems | ZMO0052 | Major facilitator superfamily protein |  |  |  | 2.03 | 2.50 |  |
| Genetic Information Processing/Environmental Information Processing/Cellular Process/Organismal Systems | ZMO0074 | Hypothetical protein | 5.02 | 3.61 |  |  |  |  |
| Genetic Information Processing/Environmental Information Processing/Cellular Process/Organismal Systems | ZMO0108 | Small multidrug resistance protein |  |  |  | 2.22 | 2.25 |  |
| Genetic Information Processing/Environmental Information Processing/Cellular Process/Organismal Systems | ZMO0209 | 50S ribosomal protein L27 |  |  |  | 2.10 | 2.51 |  |
| Genetic Information Processing/Environmental Information Processing/Cellular Process/Organismal Systems | ZMO0211 | 50S ribosomal protein L21 |  |  |  | 4.07 | 3.58 |  |
| Genetic Information Processing/Environmental Information Processing/Cellular Process/Organismal Systems | ZMO0220 | 4Fe-4S ferredoxin |  |  |  | 3.16 | 2.46 |  |
| Genetic Information Processing/Environmental Information Processing/Cellular Process/Organismal Systems | ZMO0249 | 50S ribosomal protein L33 |  |  |  | 2.19 | 2.32 |  |
| Genetic Information Processing/Environmental Information Processing/Cellular Process/Organismal Systems | ZMO0282 | RND family efflux transporter subunit MFP | 6.05 | 7.14 | 2.55 |  |  |  |
| Genetic Information Processing/Environmental Information Processing/Cellular Process/Organismal Systems | ZMO0283 | Hydrophobe/amphiphile efflux-1 (HAE1) familytransporter | 5.92 | 6.59 | 2.63 |  |  |  |
| Genetic Information Processing/Environmental Information Processing/Cellular Process/Organismal Systems | ZMO0286 | Hypothetical protein |  |  |  | 3.84 | 3.70 | 3.87 |
| Genetic Information Processing/Environmental Information Processing/Cellular Process/Organismal Systems | ZMO0412 | Multiple antibiotic resistance (MarC)-relatedprotein |  |  |  | 4.92 | 3.12 |  |
| Genetic Information Processing/Environmental Information Processing/Cellular Process/Organismal Systems | ZMO0464 | Preprotein translocase subunit SecG |  |  |  | 2.64 | 2.09 |  |
| Genetic Information Processing/Environmental Information Processing/Cellular Process/Organismal Systems | ZMO0472 | rpsU-divergently transcribed protein | 3.03 | 2.55 |  |  |  |  |
| Genetic Information Processing/Environmental Information Processing/Cellular Process/Organismal Systems | ZMO0486 | Hypothetical protein | 2.76 | 2.41 |  |  |  |  |
| Genetic Information Processing/Environmental Information Processing/Cellular Process/Organismal Systems | ZMO0494 | Drug resistance transporter, Bcr/CflA subfamily |  |  |  | 7.08 | 3.65 |  |
| Genetic Information Processing/Environmental Information Processing/Cellular Process/Organismal Systems | ZMO0495 | Hypothetical protein |  |  |  | 4.47 | 3.08 |  |
| Genetic Information Processing/Environmental Information Processing/Cellular Process/Organismal Systems | ZMO0506 | Peptidyl-tRNA hydrolase domain-containingprotein |  |  |  | 3.77 | 2.35 |  |
| Genetic Information Processing/Environmental Information Processing/Cellular Process/Organismal Systems | ZMO0546 | sulfate transporter |  |  |  | 3.57 | 2.33 |  |
| Genetic Information Processing/Environmental Information Processing/Cellular Process/Organismal Systems | ZMO0551 | tRNA pseudouridine synthase B |  |  |  | 3.28 | 2.64 |  |
| Genetic Information Processing/Environmental Information Processing/Cellular Process/Organismal Systems | ZMO0557 | Hypothetical protein |  |  |  | 3.80 | 2.27 |  |
| Genetic Information Processing/Environmental Information Processing/Cellular Process/Organismal Systems | ZMO0614 | Flagellar basal-body rod protein FlgB |  |  |  | 4.28 | 2.47 |  |
| Genetic Information Processing/Environmental Information Processing/Cellular Process/Organismal Systems | ZMO0652 | Flagellar protein FliS |  |  |  | 2.37 | 2.28 |  |
| Genetic Information Processing/Environmental Information Processing/Cellular Process/Organismal Systems | ZMO0697 | Small multidrug resistance protein |  |  |  | 3.20 | 2.87 |  |
| Genetic Information Processing/Environmental Information Processing/Cellular Process/Organismal Systems | ZMO0733 | GCN5-related N-acetyltransferase |  |  |  | 2.25 | 2.22 |  |
| Genetic Information Processing/Environmental Information Processing/Cellular Process/Organismal Systems | ZMO0750 | RluA family pseudouridine synthase |  |  |  |  | 2.40 | 3.72 |
| Genetic Information Processing/Environmental Information Processing/Cellular Process/Organismal Systems | ZMO0799 | ABC-2 type transporter | 2.35 | 3.64 | 2.17 |  |  |  |
| Genetic Information Processing/Environmental Information Processing/Cellular Process/Organismal Systems | ZMO0800 | ABC transporter | 2.23 | 3.99 | 2.19 |  |  |  |
| Genetic Information Processing/Environmental Information Processing/Cellular Process/Organismal Systems | ZMO0801 | Secretion protein HlyD family protein | 2.09 | 3.59 |  |  |  |  |
| Genetic Information Processing/Environmental Information Processing/Cellular Process/Organismal Systems | ZMO0866 | Cation diffusion facilitator family transporter |  |  |  | 4.64 | 3.12 |  |
| Genetic Information Processing/Environmental Information Processing/Cellular Process/Organismal Systems | ZMO0965 | Efflux pump membrane protein | 2.08 | 5.02 |  |  |  |  |
| Genetic Information Processing/Environmental Information Processing/Cellular Process/Organismal Systems | ZMO0979 | TonB-dependent receptor |  |  |  | 5.73 | 3.55 |  |
| Genetic Information Processing/Environmental Information Processing/Cellular Process/Organismal Systems | ZMO0998 | Peptide methionine sulfoxide reductase |  |  |  |  | 2.48 | 4.89 |
| Genetic Information Processing/Environmental Information Processing/Cellular Process/Organismal Systems | ZMO0999 | Sua5/YciO/YrdC/YwlC family protein |  |  |  | 2.78 | 2.30 | 2.76 |
| Genetic Information Processing/Environmental Information Processing/Cellular Process/Organismal Systems | ZMO1007 | Hypothetical protein |  |  |  | 2.24 |  | 3.35 |
| Genetic Information Processing/Environmental Information Processing/Cellular Process/Organismal Systems | ZMO1014 | Translation initiation factor IF-1 |  |  |  | 3.76 | 2.47 |  |
| Genetic Information Processing/Environmental Information Processing/Cellular Process/Organismal Systems | ZMO1060 | Superoxide dismutase |  |  |  |  | 2.90 | 2.78 |
| Genetic Information Processing/Environmental Information Processing/Cellular Process/Organismal Systems | ZMO1078 | tRNA (guanine-N(1)-)-methyltransferase |  |  |  | 3.28 | 2.66 |  |
| Genetic Information Processing/Environmental Information Processing/Cellular Process/Organismal Systems | ZMO1079 | 50S ribosomal protein L19 |  |  |  | 2.93 | 2.13 |  |
| Genetic Information Processing/Environmental Information Processing/Cellular Process/Organismal Systems | ZMO1136 | Cytochrome-c peroxidase |  |  |  | 3.25 | 2.15 |  |
| Genetic Information Processing/Environmental Information Processing/Cellular Process/Organismal Systems | ZMO1162 | Histidine kinase |  |  |  | 2.91 | 2.28 |  |
| Genetic Information Processing/Environmental Information Processing/Cellular Process/Organismal Systems | ZMO1174 | Surface antigen (D15) |  |  |  | 3.56 | 2.16 |  |
| Genetic Information Processing/Environmental Information Processing/Cellular Process/Organismal Systems | ZMO1246 | 50S ribosomal protein L36 |  |  |  | 2.38 | 2.56 |  |
| Genetic Information Processing/Environmental Information Processing/Cellular Process/Organismal Systems | ZMO1253 | Cytochrome C biogenesis protein | 2.76 | 2.45 |  |  |  |  |
| Genetic Information Processing/Environmental Information Processing/Cellular Process/Organismal Systems | ZMO1254 | Redoxin domain-containing protein | 2.28 | 2.12 |  |  |  |  |
| Genetic Information Processing/Environmental Information Processing/Cellular Process/Organismal Systems | ZMO1283 | LacI family transcriptional regulator |  |  |  | 2.11 | 2.62 |  |
| Genetic Information Processing/Environmental Information Processing/Cellular Process/Organismal Systems | ZMO1311 | Organic solvent tolerance protein |  |  |  | 2.64 | 2.06 |  |
| Genetic Information Processing/Environmental Information Processing/Cellular Process/Organismal Systems | ZMO1417 | DEAD/DEAH box helicase domain-containingprotein |  |  |  | 5.93 | 2.40 |  |
| Genetic Information Processing/Environmental Information Processing/Cellular Process/Organismal Systems | ZMO1527 | Acriflavin resistance protein | 2.75 | 2.11 |  |  |  |  |
| Genetic Information Processing/Environmental Information Processing/Cellular Process/Organismal Systems | ZMO1528 | Acriflavin resistance protein | 4.24 | 3.28 |  |  |  |  |
| Genetic Information Processing/Environmental Information Processing/Cellular Process/Organismal Systems | ZMO1530 | KpsF/GutQ family protein | 3.34 | 2.13 |  |  |  |  |
| Genetic Information Processing/Environmental Information Processing/Cellular Process/Organismal Systems | ZMO1579 | Alpha/beta hydrolase |  |  |  | 2.14 | 2.74 | 2.12 |
| Genetic Information Processing/Environmental Information Processing/Cellular Process/Organismal Systems | ZMO1601 | Ribonuclease H | 2.08 | 2.13 |  |  |  |  |
| Genetic Information Processing/Environmental Information Processing/Cellular Process/Organismal Systems | ZMO1625 | Ribonuclease P protein component |  |  |  | 2.17 | 2.02 |  |
| Genetic Information Processing/Environmental Information Processing/Cellular Process/Organismal Systems | ZMO1702 | DNA translocase FtsK |  |  |  | 2.08 | 2.24 |  |
| Genetic Information Processing/Environmental Information Processing/Cellular Process/Organismal Systems | ZMO1727 | Aminotransferase | 3.02 | 2.09 |  |  |  |  |
| Genetic Information Processing/Environmental Information Processing/Cellular Process/Organismal Systems | ZMO1743 | Bis(5'nucleosyl)-tetraphosphatase, ApaH |  |  |  | 2.47 | 2.23 |  |
| Genetic Information Processing/Environmental Information Processing/Cellular Process/Organismal Systems | ZMO1750 | Hypothetical protein |  |  |  | 3.95 | 4.13 | 3.09 |
| Genetic Information Processing/Environmental Information Processing/Cellular Process/Organismal Systems | ZMO1753 | Oxidoreductase FAD/NAD(P)-bindingdomain-containing protein |  |  |  |  | 2.08 | 2.10 |
| Genetic Information Processing/Environmental Information Processing/Cellular Process/Organismal Systems | ZMO1815 | TonB-dependent siderophore receptor |  |  |  | 6.58 | 6.08 | 4.38 |
| Genetic Information Processing/Environmental Information Processing/Cellular Process/Organismal Systems | ZMO1836 | Nitrogen fixation protein NifW |  |  |  | 2.52 | 2.92 |  |
| Genetic Information Processing/Environmental Information Processing/Cellular Process/Organismal Systems | ZMO1847 | Transport system permease |  |  |  |  | 2.78 | 2.11 |
| Genetic Information Processing/Environmental Information Processing/Cellular Process/Organismal Systems | ZMO1854 | GntR family transcriptional regulator |  |  |  |  | 2.40 | 2.29 |
| Genetic Information Processing/Environmental Information Processing/Cellular Process/Organismal Systems | ZMO1988 | tRNA modification GTPase TrmE |  |  |  | 2.26 | 2.24 |  |
| Genetic Information Processing/Environmental Information Processing/Cellular Process/Organismal Systems | ZMOt037 | tRNA-Met |  |  |  | 2.26 | 2.05 |  |
| Genetic Information Processing/Environmental Information Processing/Cellular Process/Organismal Systems | ZMOt038 | tRNA-Ala |  |  |  | 2.39 | 2.27 |  |
| Genetic Information Processing/Environmental Information Processing/Cellular Process/Organismal Systems | ZMOt039 | tRNA-Ile |  |  |  | 2.41 | 2.17 |  |
| Genetic Information Processing/Environmental Information Processing/Cellular Process/Organismal Systems | ZMOt040 | tRNA-Lys |  |  |  | 2.96 | 3.59 |  |
| Lipid Metabolism | ZMO1222 | 3-oxoacyl-ACP reductase | 2.73 | 2.11 |  |  |  |  |
| Metabolism of Cofactors and Vitamins | ZMO0006 | Uroporphyrin-III C-methyltransferase | 3.09 | 2.05 |  |  |  |  |
| Metabolism of Cofactors and Vitamins | ZMO0172 | Thiamine biosynthesis protein ThiC |  |  |  | 3.41 | 3.45 |  |
| Metabolism of Cofactors and Vitamins | ZMO0474 | 3,4-dihydroxy-2-butanone 4-phosphate synthase | 2.71 | 2.34 |  |  |  |  |
| Metabolism of Cofactors and Vitamins | ZMO1006 | Dihydropteroate synthase |  |  |  | 4.17 | 3.39 |  |
| Metabolism of Cofactors and Vitamins | ZMO1132 | Lipoyl synthase | 2.17 | 2.33 |  |  |  |  |
| Metabolism of Cofactors and Vitamins | ZMO1190 | Bifunctional phosphopantothenoylcysteinedecarboxylase /phosphopantothenate synthase | 2.42 | 2.41 |  |  |  |  |
| Metabolism of Cofactors and Vitamins | ZMO1544 | Cobalt chelatase, pCobS small subunit | 2.84 | 2.01 |  |  |  |  |
| Metabolism of Cofactors and Vitamins | ZMO1586 | Bacterioferritin |  |  |  |  | 2.46 | 2.47 |
| Metabolism of Other Amino acids | ZMO0002 | Class I and II aminotransferase |  |  |  | 2.14 | 2.26 |  |
| Metabolism of Other Amino acids | ZMO1118 | Glutathione S-transferase domain-containingprotein |  |  |  | 2.80 | 2.54 |  |
| Metabolism of Other Amino acids | ZMO1309 | Leucyl aminopeptidase | 2.33 | 2.03 |  |  |  |  |
| Metabolism of Other Amino acids | ZMO1776 | Peptidase M1 membrane alanine aminopeptidase |  |  |  | 3.33 |  | 3.64 |
| Nucleotide Metabolism | ZMO0655 | Adenosine/AMP deaminase |  |  |  | 2.97 | 2.05 |  |
| Nucleotide Metabolism | ZMO1321 | Inosine-5'-monophosphate dehydrogenase |  |  |  | 3.58 | 2.08 |  |
| Nucleotide Metabolism | ZMO1720 | DNA-directed RNA polymerase subunit omega |  |  |  | 2.59 | 2.43 |  |
| Unassigned | ZMO0020 | Hypothetical protein | 3.08 | 2.14 | 2.36 |  |  |  |
| Unassigned | ZMO0021 | Hypothetical protein | 3.11 | 2.89 | 2.38 |  |  |  |
| Unassigned | ZMO0026 | Sel1 domain-containing protein repeat-containingprotein |  |  |  | 4.88 | 3.41 | 2.43 |
| Unassigned | ZMO0038 | Sigma 54 modulation protein/ribosomal proteinS30EA |  |  |  |  | 3.50 | 2.25 |
| Unassigned | ZMO0073 | CBS domain-containing protein | 5.98 | 4.13 |  |  |  |  |
| Unassigned | ZMO0089 | Hypothetical protein |  |  |  | 2.09 | 2.66 |  |
| Unassigned | ZMO0090 | Short-chain dehydrogenase/reductase SDR |  |  |  | 2.27 | 2.11 | 2.22 |
| Unassigned | ZMO0095 | Hypothetical protein |  |  |  | 9.68 | 5.91 |  |
| Unassigned | ZMO0109 | Hypothetical protein |  |  |  | 2.68 | 2.68 |  |
| Unassigned | ZMO0122 | Hypothetical protein |  |  |  |  | 2.27 | 2.92 |
| Unassigned | ZMO0157 | D-isomer specific 2-hydroxyacid dehydrogenaseNAD-binding protein | 2.28 | 2.22 |  |  |  |  |
| Unassigned | ZMO0184 | Hypothetical protein |  |  |  | 4.46 | 3.22 |  |
| Unassigned | ZMO0190 | RpiR family transcriptional regulator |  |  |  | 5.17 | 4.28 | 2.18 |
| Unassigned | ZMO0204 | Cation efflux protein |  |  |  | 10.55 | 9.77 |  |
| Unassigned | ZMO0205 | NAD-dependent epimerase/dehydratase |  |  |  | 2.52 | 3.23 |  |
| Unassigned | ZMO0208 | GCN5-related N-acetyltransferase |  |  |  |  | 2.16 | 2.40 |
| Unassigned | ZMO0268 | Hypothetical protein | 2.32 | 2.7 |  |  |  |  |
| Unassigned | ZMO0270 | Hypothetical protein | 2.41 | 2.91 |  |  |  |  |
| Unassigned | ZMO0293 | Sugar transporter |  |  |  | 5.25 | 2.44 |  |
| Unassigned | ZMO0315 | Hypothetical protein |  |  |  | 2.20 | 2.38 |  |
| Unassigned | ZMO0331 | Hypothetical protein |  |  |  | 2.06 | 2.13 |  |
| Unassigned | ZMO0384 | Hypothetical protein |  | 2.89 | 2.07 |  |  |  |
| Unassigned | ZMO0388 | Hypothetical protein |  | 2.30 | 2.15 |  |  |  |
| Unassigned | ZMO0391 | Hypothetical protein |  | 2.76 | 2.22 |  |  |  |
| Unassigned | ZMO0422 | BadM/Rrf2 family transcriptional regulator |  |  |  | 2.08 | 2.21 |  |
| Unassigned | ZMO0440 | Hypothetical protein | 2.25 | 2.36 |  |  |  |  |
| Unassigned | ZMO0501 | GtrA family protein |  |  |  | 3.01 | 2.08 |  |
| Unassigned | ZMO0683 | Csy2 family CRISPR-associated protein | 2.55 | 2.47 |  |  |  |  |
| Unassigned | ZMO0686 | Signal transduction protein |  |  |  | 3.69 | 2.21 |  |
| Unassigned | ZMO0693 | OsmC family protein |  |  |  |  | 2.24 | 2.87 |
| Unassigned | ZMO0695 | Hypothetical protein |  |  |  | 3.76 | 2.84 |  |
| Unassigned | ZMO0740 | CsbD family protein |  |  |  |  | 2.00 | 2.24 |
| Unassigned | ZMO0757 | TPR repeat-containing protein | 2.02 | 2.92 |  |  |  |  |
| Unassigned | ZMO0758 | Isochorismatase hydrolase | 2.41 | 2.55 |  |  |  |  |
| Unassigned | ZMO0763 | Tetratricopeptide domain-containing protein | 2.48 | 2.01 |  |  |  |  |
| Unassigned | ZMO0795 | TonB-dependent receptor plug |  |  |  | 3.39 | 2.88 |  |
| Unassigned | ZMO0798 | NodT family RND efflux system outer membranelipoprotein | 2.27 | 4.58 | 2.54 |  |  |  |
| Unassigned | ZMO0844 | Sporulation domain-containing protein | 2.32 | 2.09 |  |  |  |  |
| Unassigned | ZMO0936 | ypothetical protein |  |  |  | 2.74 | 2.2 |  |
| Unassigned | ZMO0955 | --- |  |  |  | 2.19 | 2.25 |  |
| Unassigned | ZMO0996 | Mechanosensitive ion channel MscS |  |  |  | 3.35 | 2.86 |  |
| Unassigned | ZMO1045 | Phosphate-selective porin O and P |  |  |  | 3.52 | 2.71 |  |
| Unassigned | ZMO1057 | GCN5-related N-acetyltransferase |  |  |  | 2.57 | 2.35 |  |
| Unassigned | ZMO1080 | Hypothetical protein |  |  |  | 7.22 | 3.72 |  |
| Unassigned | ZMO1081 | Hypothetical protein |  |  |  | 4.70 | 2.34 |  |
| Unassigned | ZMO1082 | Hypothetical protein |  |  |  | 5.66 | 2.51 |  |
| Unassigned | ZMO1083 | Cellulose synthase catalytic subunit |  |  |  | 3.81 | 2.10 |  |
| Unassigned | ZMO1144 | VanZ family protein |  |  |  | 3.91 | 2.34 |  |
| Unassigned | ZMO1215 | Hypothetical protein |  |  |  | 2.32 | 2.85 | 3.08 |
| Unassigned | ZMO1249 | Hypothetical protein |  |  |  | 3.47 | 2.13 |  |
| Unassigned | ZMO1288 | Major facilitator superfamily protein |  |  |  | 4.07 | 3.44 | 4.18 |
| Unassigned | ZMO1289 | Transglycosylase-associated protein |  |  |  | 4.19 | 5.64 | 4.58 |
| Unassigned | ZMO1334 | Hypothetical protein | 4.11 | 2.96 |  |  |  |  |
| Unassigned | ZMO1346 | Hypothetical protein | 2.23 | 2.39 |  |  |  |  |
| Unassigned | ZMO1354 | OmpA/MotB domain-containing protein |  |  |  | 2.44 | 2.05 |  |
| Unassigned | ZMO1377 | Hypothetical protein |  |  |  | 2.45 | 2.07 |  |
| Unassigned | ZMO1380 | AraC family transcriptional regulator | 5.17 | 2.60 | 2.21 |  |  |  |
| Unassigned | ZMO1386 | Hypothetical protein | 3.44 | 2.39 |  |  |  |  |
| Unassigned | ZMO1391 | Diacylglycerol kinase catalytic region | 2.42 | 2.69 |  |  |  |  |
| Unassigned | ZMO1399 | Fatty acid hydroxylase | 3.94 | 2.98 |  |  |  |  |
| Unassigned | ZMO1406 | Alpha/beta hydrolase | 3.94 | 2.26 |  |  |  |  |
| Unassigned | ZMO1437 | Lysine exporter protein | 3.72 | 2.42 | 2.81 |  |  |  |
| Unassigned | ZMO1456 | Lysine exporter protein |  |  |  | 6.10 | 3.59 |  |
| Unassigned | ZMO1457 | Major facilitator superfamily protein |  |  |  | 5.27 | 4.24 |  |
| Unassigned | ZMO1511 | Hypothetical protein |  |  |  | 4.63 | 3.40 |  |
| Unassigned | ZMO1521 | hypothetical protein |  |  |  | 4.29 | 3.91 |  |
| Unassigned | ZMO1529 | RND family efflux transporter subunit MFP | 2.54 | 2.32 |  |  |  |  |
| Unassigned | ZMO1535 | --- |  |  |  | 3.29 | 2.70 |  |
| Unassigned | ZMO1574 | LysR family transcriptional regulator |  |  |  | 3.51 | 2.30 | 2.01 |
| Unassigned | ZMO1576 | Short-chain dehydrogenase/reductase SDR | 3.77 | 2.17 |  |  |  |  |
| Unassigned | ZMO1577 | Major facilitator superfamily protein |  |  |  |  | 2.97 | 2.93 |
| Unassigned | ZMO1578 | Short-chain dehydrogenase/reductase SDR |  |  |  |  | 2.26 | 2.19 |
| Unassigned | ZMO1602 | Hypothetical protein | 2.77 | 2.17 |  |  |  |  |
| Unassigned | ZMO1620 | Hypothetical protein |  |  |  | 9.25 | 2.57 |  |
| Unassigned | ZMO1696 | Zinc-binding alcohol dehydrogenase | 2.46 | 2.94 |  |  |  |  |
| Unassigned | ZMO1784 | Hypothetical protein |  |  |  | 2.54 | 2.54 |  |
| Unassigned | ZMO1785 | Mechanosensitive ion channel MscS |  |  |  | 2.92 | 2.22 |  |
| Unassigned | ZMO1794 | Hypothetical protein |  |  |  | 2.71 | 2.60 |  |
| Unassigned | ZMO1821 | Hypothetical protein | 3.62 | 4.26 |  |  |  |  |
| Unassigned | ZMO1856 | Major facilitator superfamily transporter |  |  |  | 4.40 | 3.30 | 2.44 |
| Unassigned | ZMO1857 | LysR family transcriptional regulator |  |  |  | 2.47 | 3.83 | 2.67 |
| Unassigned | ZMO1864 | Transposase IS5 family protein |  |  |  | 2.65 | 3.60 | 2.64 |
| Unassigned | ZMO1880 | Hypothetical protein | 2.58 | 2.90 |  |  |  |  |
| Unassigned | ZMO1885 | NADH:flavin oxidoreductase/NADH oxidase | 11.37 | 3.33 | 2.67 |  |  |  |
| Unassigned | ZMO1932 | Hypothetical protein |  |  |  | 2.53 | 2.50 |  |
| Unassigned | ZMO1976 | Hypothetical protein |  |  |  | 3.22 | 3.34 |  |
| Unassigned | ZMO1984 | Aldo/keto reductase | 3.87 | 2.06 |  |  |  |  |
| Unassigned | ZMO1986 | TonB-dependent receptor plug |  |  |  | 2.84 | 2.67 |  |
| Unassigned | ZMO2000 | --- |  |  |  | 3.44 | 3.22 |  |
| Unassigned | ZMO2006 | Preprotein translocase subunit SecE |  |  |  | 2.14 | 2.10 |  |
| Unassigned | ZMO2013 | Hypothetical protein |  |  |  | 3.89 | 2.40 |  |
| Unassigned | ZMO2016 | Hypothetical protein |  |  |  | 3.75 | 2.44 |  |
| Unassigned | ZMO2020 | Hypothetical protein |  |  |  | 5.97 | 4.02 |  |
| Unassigned | ZMO2030 | Hypothetical protein |  |  |  | 20.40 | 12.44 | 4.06 |
| Unassigned | ZMO2034 | Hypothetical protein |  |  |  | 2.03 | 2.97 |  |
|  | ZZM4_0002 | Addiction module antitoxin, RelB/DinJ family |  |  |  | 3.05 | 2.59 |  |
|  | ZZM4_0003 | Addiction module toxin, RelE/StbE family |  |  |  | 2.85 | 3.83 |  |
|  | ZZM4_0004 | --- |  |  |  | 4.70 | 2.56 |  |
|  | ZZM4_0005 | Hypothetical protein |  |  |  | 5.20 | 4.49 |  |
|  | ZZM4_0008 | Hypothetical protein |  |  |  | 4.59 | 3.27 |  |
|  | ZZM4_0009 | Hypothetical protein |  |  |  | 4.61 | 2.91 |  |
|  | ZZM4_0011 | Hypothetical protein |  |  |  | 5.85 | 2.56 |  |
|  | ZZM4_0022 | Hypothetical protein |  |  |  | 2.13 | 2.13 |  |
|  | ZZM4_0023 | Phage tail protein I |  |  |  | 2.59 | 2.17 |  |
|  | ZZM4_0026 | Hypothetical protein |  |  |  | 2.30 | 2.19 |  |
|  | ZZM4_0027 | Hypothetical protein |  |  |  | 4.74 | 3.06 |  |
|  | ZZM4_0028 | Hypothetical protein |  |  |  | 2.36 | 2.21 |  |
|  | ZZM4_0042 | Protein of unknown function DUF1526 |  |  |  | 2.69 | 2.42 |  |
|  | ZZM4_0043 | Hypothetical protein |  |  |  | 6.58 | 3.12 |  |
|  | ZZM4_0044 | Hypothetical protein |  |  |  | 18.00 | 4.92 |  |
|  | ZZM4_0046 | Transcriptional activator Ogr/delta |  |  |  | 29.12 | 5.86 |  |
|  | ZZM4_0068 | Typothetical protein |  |  |  | 4.84 | 7.37 | 2.73 |
|  | ZZM4_0069 | Hypothetical protein |  |  |  | 2.99 | 3.76 | 2.40 |
|  | ZZM4_0070 | Hypothetical protein |  |  |  | 2.50 | 2.75 | 2.30 |
|  | ZZM4_0071 | Hypothetical protein |  |  |  | 2.77 | 2.66 |  |
|  | ZZM4_0081 | Hypothetical protein |  |  |  | 5.26 | 2.32 |  |
|  | ZZM4_0101 | Hypothetical protein |  |  |  | 2.13 | 2.68 | 2.26 |
|  | ZZM4_0102 | Hhypothetical protein |  |  |  | 2.17 | 2.27 | 2.29 |
|  | ZZM4_0103 | Hypothetical protein |  |  |  | 2.34 | 2.45 |  |
|  | ZZM4_0110 | Hypothetical protein |  |  |  | 4.19 | 2.40 |  |
|  | ZZM4_0117 | Hypothetical protein |  |  |  | 4.59 | 2.26 |  |
|  | ZZM4_0119 | Hhypothetical protein |  |  |  | 3.77 | 6.64 | 2.49 |
|  | ZZM4_0120 | Hypothetical protein |  |  |  | 2.40 | 2.60 | 2.35 |
|  | ZZM4_0122 | Hypothetical protein |  |  |  | 4.57 | 3.06 |  |
|  | ZZM4_0133 | Hypothetical protein | 2.39 | 2.75 |  |  |  |  |
|  | ZZM4_0167 | Hypothetical protein |  |  |  | 2.61 | 2.73 | 2.34 |
|  | ZZM4_0168 | Hypothetical protein |  |  |  |  | 2.54 | 2.05 |
|  | ZZM4_0169 | Hypothetical protein |  |  |  | 2.26 | 2.50 | 2.10 |
|  | pzmob1_p06 | Hypothetical protein |  |  |  | 6.13 | 3.26 |  |
|  | pzmob1_p07 | Hypothetical protein |  |  |  | 11.91 | 3.26 |  |
|  | pzmob1_p11 | Hypothetical protein |  |  |  | 4.34 | 6.99 | 2.66 |
|  | pzmob1_p12 | Hypothetical protein |  |  |  | 3.42 | 3.75 | 2.50 |
|  | pzmob1_p13 | Hypothetical protein |  |  |  | 2.61 | 2.75 | 2.32 |
|  | pzmob1_p14 | Hypothetical protein |  |  |  | 2.04 | 2.14 | 2.07 |
|  | pzmob1_p16 | Hypothetical protein |  |  |  | 4.02 | 2.29 |  |
|  | pzmob1_p18 | Hypothetical protein |  |  |  | 2.35 | 2.03 |  |
|  | pzmob1_p21 | Hypothetical protein |  |  |  | 7.10 | 2.78 |  |
|  | pzmob1_p33 | Hypothetical protein |  |  |  | 3.58 | 2.42 |  |
|  | pzmob1_p38 | Hypothetical protein | 2.29 | 2.02 |  |  |  |  |
